# Complementarity of neutron, XFEL and synchrotron crystallography for defining the structures of metalloenzymes at room temperature

**DOI:** 10.1107/S2052252522006418

**Published:** 2022-07-25

**Authors:** Tadeo Moreno-Chicano, Leiah M. Carey, Danny Axford, John H. Beale, R. Bruce Doak, Helen M. E. Duyvesteyn, Ali Ebrahim, Robert W. Henning, Diana C. F. Monteiro, Dean A. Myles, Shigeki Owada, Darren A. Sherrell, Megan L. Straw, Vukica Šrajer, Hiroshi Sugimoto, Kensuke Tono, Takehiko Tosha, Ivo Tews, Martin Trebbin, Richard W. Strange, Kevin L. Weiss, Jonathan A. R. Worrall, Flora Meilleur, Robin L. Owen, Reza A. Ghiladi, Michael A. Hough

**Affiliations:** a School of Life Sciences, University of Essex, Wivenhoe Park, Colchester CO4 3SQ, United Kingdom; b Department of Chemistry, North Carolina State University, Raleigh, NC 27695-8204, USA; c Diamond Light Source, Harwell Science and Innovation Campus, Didcot OX11 0DE, United Kingdom; d Max Planck Institute for Medical Research, Heidelberg, Germany; e Division of Structural Biology (STRUBI), University of Oxford, The Henry Wellcome Building for Genomic Medicine, Roosevelt Drive, Oxford OX3 7BN, United Kingdom; f BioCARS, University of Chicago, Building 434B, Argonne National Laboratory, 9700 South Cass Avenue, Lemont, IL 60439, USA; g Hauptman–Woodward Medical Research Institute, 700 Ellicott Street, Buffalo, NY 14203-1102, USA; h Oak Ridge National Laboratory, Oak Ridge, Tennessee, USA; i Japan Synchrotron Radiation Research Institute, 1-1-1 Kouto, Sayo, Hyogo 679-5198, Japan; j Structural Biology Center, X-ray Science Division, Argonne National Laboratory, Argonne, IL 60439, USA; k RIKEN SPring-8 Center, 1-1-1 Kouto, Sayo, Hyogo 679-5198, Japan; l Biological Sciences, University of Southampton, University Road, Southampton SO17 1BJ, United Kingdom; m Department of Chemistry, State University of New York at Buffalo, Buffalo, NY 14260, USA; King’s College London, United Kingdom and University of Padova, Italy

**Keywords:** serial femtosecond crystallography, serial synchrotron crystallography, neutron crystallography, XFELs, room temperature, metalloenzymes

## Abstract

Room-temperature macromolecular crystallography allows protein structures to be determined under close-to-physiological conditions, permits dynamic freedom in protein motions and enables time-resolved studies. In the case of metalloenzymes that are highly sensitive to radiation damage, such room-temperature experiments can present challenges, including increased rates of X-ray reduction of metal centres and site-specific radiation-damage artefacts, as well as in devising appropriate sample-delivery and data-collection methods. It can also be problematic to compare structures measured using different crystal sizes and light sources. In this study, structures of a multifunctional globin, dehaloperoxidase B (DHP-B), obtained using several methods of room-temperature crystallographic structure determination are described and compared. Here, data were measured from large single crystals and multiple microcrystals using neutrons, X-ray free-electron laser pulses, monochromatic synchrotron radiation and polychromatic (Laue) radiation light sources. These approaches span a range of 18 orders of magnitude in measurement time per diffraction pattern and four orders of magnitude in crystal volume. The first room-temperature neutron structures of DHP-B are also presented, allowing the explicit identification of the hydrogen positions. The neutron data proved to be complementary to the serial femtosecond crystallography data, with both methods providing structures free of the effects of X-ray radiation damage when compared with standard cryo-crystallography. Comparison of these room-temperature methods demonstrated the large differences in sample requirements, data-collection time and the potential for radiation damage between them. With regard to the structure and function of DHP-B, despite the results being partly limited by differences in the underlying structures, new information was gained on the protonation states of active-site residues which may guide future studies of DHP-B.

## Abstract 2

The determination of the structure of the multifunctional globin dehaloperoxidase using multiple room-temperature methods is described. Structures obtained by serial femtosecond crystallography, serial synchrotron crystallography, neutron diffraction and serial Laue crystallography are compared and two oxidation states of the enzyme are contrasted.

## 1. Introduction

The importance of crystal structures determined at ambient, or room temperature (RT), for understanding protein function and dynamics has recently become increasingly recognized (Keedy *et al.*, 2018 ▸). Crystalline proteins at RT exhibit greater conformational freedom and avoid the increase in mosaic spread often caused by cryocooling and/or the addition of cryoprotectants (Russi *et al.*, 2017 ▸). Moreover, recent studies have revealed significant alterations to side-chain conformations and ligand binding with temperature (Gerlits *et al.*, 2017 ▸; Fischer *et al.*, 2015 ▸; Atakisi *et al.*, 2018 ▸; Keedy *et al.*, 2018 ▸). As such, there has been great interest in experimental methods that allow RT protein structures to be obtained to high resolution (Helliwell, 2020*a* ▸; Fischer, 2021 ▸).

However, the ability to obtain a sufficiently complete room-temperature data set for structure determination using X-rays is greatly limited by the two orders of magnitude reduction in crystal dose lifetime in RT crystals compared with those cryocooled to 100 K (Southworth-Davies *et al.*, 2007 ▸; Holton, 2009 ▸). The challenge is particularly acute for metalloproteins, such as heme enzymes, where X-ray-induced changes to the metal-containing active site occur even at extremely low doses and cryogenic temperatures (Beitlich *et al.*, 2007 ▸; Kekilli *et al.*, 2017 ▸; Pfanzagl *et al.*, 2020 ▸). The vast majority of X-rays that interact with a protein crystal do so *via* the photoelectric effect and result in ionization, radiolysis of water and further secondary events that lead to several hundred solvated electrons being generated per absorbed X-ray photon (Holton, 2009 ▸). This can rapidly cause alterations in the ionization state of metalloprotein active sites before more general, global, damage to the protein structure and crystal lattice.

Attempts to obtain RT structures free of radiation damage have largely focused on two approaches, neutron crystallo­graphy (NX) and serial femtosecond crystallography (SFX), in which the ‘diffraction before destruction’ principle allows damage-free structures to be determined (Neutze *et al.*, 2000 ▸; Helliwell, 2020*b* ▸). These approaches span the extremes of macromolecular crystal size, from microcrystal (tens of micrometres or smaller) dimensions (SFX) to millimetre-sized crystals (NX). In SFX, extremely intense X-ray pulses on the scale of tens of femtoseconds are used. The crystal is destroyed by the tremendously high X-ray dose, but this occurs subsequent to the diffraction event and has either no or a very limited effect on the data, provided that the pulse is short enough (Schlichting, 2015 ▸; Neutze *et al.*, 2000 ▸; Nass *et al.*, 2020 ▸; Dickerson *et al.*, 2020 ▸). For this reason, in almost all cases SFX is carried out using many thousands of microcrystals, with each X-ray pulse contributing to the data set interacting with a fresh crystal (Schlichting, 2015 ▸). Therefore, SFX offers the possibility of collecting high-resolution X-ray diffraction data from crystals at RT without potential artefacts arising from the damaging effects of the X-ray beam.

Neutron scattering from the crystal lattice does not lead to radiation damage (O’Dell *et al.*, 2016 ▸, 2017 ▸); in neutron crystallography (NX) data are typically obtained from a single large crystal over several days of data collection. NX also has the crucial advantage that the positions of H atoms are well defined, allowing the protonation states of mechanistically important residues to be explicitly identified (Schröder *et al.*, 2018 ▸; Kwon *et al.*, 2016 ▸). In many cases, NX structures are accompanied by RT X-ray diffraction data subsequently obtained from the same crystal using laboratory X-ray sources (Knihtila *et al.*, 2015 ▸; Schröder *et al.*, 2018 ▸; Golden *et al.*, 2017 ▸), or from a different crystal prepared in a similar manner (Weber *et al.*, 2013 ▸; Kwon *et al.*, 2017 ▸). These RT X-ray data are inevitably prone to radiation damage and, in the case of metalloproteins, may not be representative of the state characterized by neutrons (Weik & Colletier, 2010 ▸).

Parallel developments have used single synchrotron X-ray exposures of microcrystals in the range of hundreds of picoseconds in Laue crystallography (Meents *et al.*, 2017 ▸) to tens of milliseconds using monochromatic synchrotron beams (Stellato *et al.*, 2014 ▸; Monteiro *et al.*, 2019 ▸, 2020 ▸; Schulz *et al.*, 2018 ▸; Mehrabi *et al.*, 2019 ▸; Oghbaey *et al.*, 2016 ▸; Sherrell *et al.*, 2015 ▸; Owen *et al.*, 2017 ▸), with the aim of mitigating the radiation damage present in the overall data set (Owen *et al.*, 2017 ▸; Sherrell *et al.*, 2015 ▸; Oghbaey *et al.*, 2016 ▸; Ebrahim, Appleby *et al.*, 2019 ▸; Ebrahim, Moreno-Chicano *et al.*, 2019 ▸). While both ‘large crystal’ and ‘microcrystal’ based methodologies have developed rapidly, there are few direct comparisons of both the practicalities and the resulting structures.

Peroxidases and other heme enzymes are important targets for ‘damage-free’ methods due to their high sensitivity to changes to their electronic and structural states in response to X-ray exposure during crystallographic data collection (Meharenna *et al.*, 2008 ▸, 2010 ▸; Moody & Raven, 2018 ▸; Ebrahim, Moreno-Chicano *et al.*, 2019 ▸; Kekilli *et al.*, 2017 ▸; Lučić *et al.*, 2021 ▸). Despite this interest, very few damage-free heme-protein structures have successfully been determined to date. NX peroxidase structures determined at 100 K include those of the ferric [Fe(III)] and ferryl [Fe(IV)=O] states of cytochrome *c* peroxidase (Casadei *et al.*, 2014 ▸), as well as that of compound II ([Fe^IV^-OH]) of soybean ascorbate peroxidase (Kwon *et al.*, 2016 ▸). There are also SFX structures at 100 K of a Fe(IV)=O form of cytochrome *c* peroxidase (Chreifi *et al.*, 2016 ▸), compound II of ascorbate peroxidase and cytochrome *c* peroxidase (Kwon *et al.*, 2021 ▸), and several RT SFX structures of the Fe(III) and Fe(IV) states of peroxidases (Ebrahim, Moreno-Chicano *et al.*, 2019 ▸; Moreno-Chicano *et al.*, 2019 ▸; Lučić *et al.*, 2020 ▸).

Our model protein for comparing methods of RT structure determination is the coelomic hemoglobin from the marine annelid *Amphitrite ornata*. Named dehaloperoxidase (DHP; Chen *et al.*, 1996 ▸; Zhang *et al.*, 1996 ▸), this O_2_-transport protein (Weber *et al.*, 1977 ▸; Sun *et al.*, 2014 ▸) is capable of oxidizing a wide array of substrates by either electron or O-atom transfer. These substrates include mono-, di- and trihalophenols (Chen *et al.*, 1996 ▸), haloindoles (Barrios *et al.*, 2014 ▸), pyrroles (McCombs, Smirnova *et al.*, 2017 ▸), (halo)guaiacols (McGuire *et al.*, 2018 ▸), nitrophenols (McCombs *et al.*, 2016 ▸) and cresols (Malewschik *et al.*, 2019 ▸). DHP can also strongly bind azoles (McCombs, Moreno-Chicano *et al.*, 2017 ▸). DHP exhibits four activities common to heme proteins, peroxidase, peroxy­gen­ase, oxidase and oxygenase, with each activity employing an Fe(IV)-oxo (ferryl) intermediate.

In this study, we aimed to compare structures of DHP obtained using multiple room-temperature methods, with the goal of understanding the advantages and disadvantages of each approach as well as the ability of each method to reveal the structure and function of this multifunctional enzyme. We describe damage-free SFX and neutron crystal structures of dehaloperoxidase isoenzyme B (DHP-B). These are denoted here as data sets SFX and NX, respectively (Table 1 ▸). To our knowledge, this is the first comparison of damage-free structures obtained through neutron crystallography and serial femtosecond crystallography *at room temperature* from any metalloprotein system. As well as this, we present an X-ray crystal structure that was determined from the same crystal as previously used for collection of the neutron data (data set NX-Xray). We also present and compare RT structures obtained by serial synchrotron crystallography (data set SSX) at a monochromatic synchrotron microfocus beamline and by serial Laue crystallography (data set SLX) using a polychromatic (pink) beam and a novel fixed-target sample-delivery system. In most of the DHP ferric structures, a ‘hemichrome’ feature is present; these are bis-His hexacoordinated low-spin heme species that have been well characterized in hemo­globins (Riccio *et al.*, 2002 ▸).

Finally, we describe structures of oxyferrous DHP-B determined by neutron and SFX methods. Our data allow us to examine and compare RT crystal structures of DHP-B produced from crystals of very different sizes [from large single crystals to microcrystals (tens of micrometres)] and measured using a variety of sources, together with the radiation-damage effects that the different experimental conditions might have on the metalloenzyme in each case.

## 2. Materials and methods

### 2.1. Protein production

#### 2.1.1. Adaptation of *Escherichia coli* to deuterated culture medium

The pET-28a plasmid containing the *dhpB* gene was transformed into *E. coli* BL21(DE3) competent cells (Novagen). All media contained kanamycin (50 µg ml^−1^) prepared in either H_2_O (DHP-B) or 99.8% D_2_O (perdeuterated DHP-B). Enfors Minimal Medium was used (protiated or deuterated; Meilleur *et al.*, 2009 ▸). In the case of 99.8% deuterated minimal media, all hydrated salts were dissolved in D_2_O and then dried *in vacuo* via rotary evaporation. This was performed twice in an effort to replace the exchangeable protons with deuterons.

Cell culture in perdeuterated media was achieved from a starting protiated culture using a stepwise approach from 0 to 99.8% D_2_O minimal media, with incubation between 28 and 37°C at different stages and orbital shaking at 250 rev min^−1^. A resulting 140 ml culture of 99.8% deuterated minimal medium was used to inoculate a 1.4 l fermenter culture.

#### 2.1.2. Bioreactor fermentation

A BioFlo 310 Fermentation System (New Brunswick Scientific) enabled the monitoring and control of pD, dissolved O_2_, temperature, agitation and carbon-source availability. The growth and expression were kept at a constant temperature of 32°C. Upon inoculation of the fermenter vessel (OD_600_ = 0.21), 10 ml 99.8% deuterated hemin stock solution (10 mg ml^−1^ in 0.2 *M* NaOD) was added to the medium. Upon reaching an OD_600_ of 9.8, overexpression of DHP-B was induced by the addition of 1.5 ml 750 m*M* 99.8% isopropyl β-d-1-thiogalactopyranoside stock solution. Overexpression continued for 20 h and the cells were harvested at an OD_600_ of 12.6 *via* centrifugation.

#### 2.1.3. Crystal growth and manipulation

Perdeuterated DHP-B was purified as described previously (de Serrano *et al.*, 2010 ▸) with only two modifications: a linear pH gradient over 20 ml was used to initiate elution from the CM Sepharose FF column and a gentler concentration of the samples was necessary to prevent precipitation of the perdeuterated protein. Homogenous ferric enzyme was obtained by incubation with excess potassium ferricyanide, which was subsequently removed using a Sephadex G-25 desalting column. Perdeuterated DHP-B was exchanged into 20 m*M* sodium cacodylate buffer pH 6.4 and then concentrated to 12 mg ml^−1^ (740 µ*M*) as determined spectroscopically, utilizing a molar absorptivity coefficient ɛ of 116 400 *M* ^−1^ cm^−1^ (D’Antonio *et al.*, 2010 ▸). MALDI analysis confirmed the successful perdeuteration of DHP-B, resulting in a 97% deuteration level (data not shown). Oxyferrous DHP-B crystals were obtained from protiated protein grown in LB medium. Purification and crystal-growth conditions were identical to those used for perdeuterated ferric DHP-B. To obtain oxyferrous DHP-B crystals, ferric crystals were incubated in mother liquor containing 10 m*M* ascorbate for a minimum of 30 min prior to D_2_O exchange.

#### 2.1.4. NX crystallization and sample preparation

**Figure 1:**
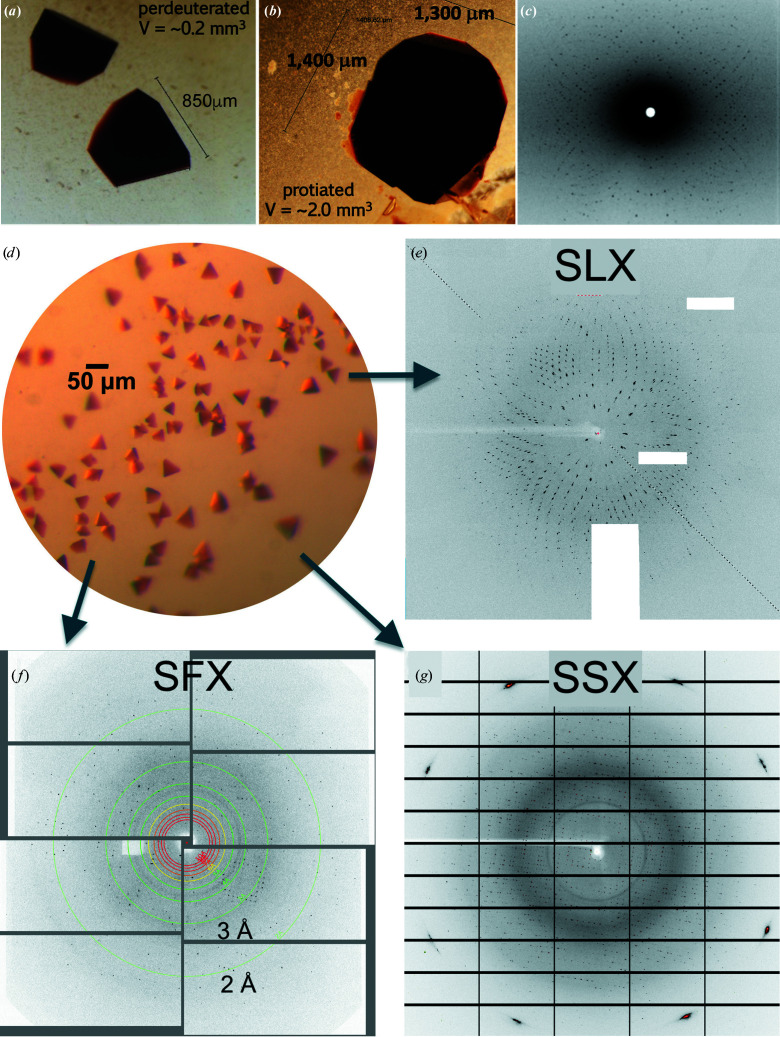
DHP-B crystals and diffraction patterns. (*a*) Perdeuterated DHP-B crystal grown in MPEG 2000, crystal volume ∼0.3 mm^3^. (*b*) Protiated DHP-B crystal grown in PEG 4000, crystal volume ∼2.0 mm^3^. (*c*) Representative quasi-Laue neutron diffraction pattern of ferric DHP-B measured at ORNL. The image was obtained from a 20 h neutron exposure. (*d*) Typical DHP-B microcrystals grown in batch mode (longest dimension 15–50 µm). (*e*) Image obtained from microcrystals by Laue diffraction at the APS BioCARS beamline. (*f*) Diffraction image obtained from microcrystals by SFX at SACLA. (*g*) Diffraction image from the SSX data measured on beamline I24 at Diamond Light Source using monochromatic X-rays.

Crystals were grown via sitting-drop vapour diffusion in nine-well siliconized glass plates in a sandwich-box setup. The mother liquor was 32%(*w*/*v*) MPEG 2000, 175 m*M* ammonium sulfate (with no additional buffering agents) and the drop ratios of protein to mother liquor were 1.5:1, 2:1, 2.5:1 and 3:1. The drops had a total volume of 100 µl and were equilibrated against 25 ml reservoir solution. Crystals appeared after six weeks at 4°C. The crystals were D_2_O-exchanged by soaking in a deuterated mother-liquor equivalent after exchanging the reservoir. Small aliquots of drop solution were replaced with deuterated reservoir. The drop was allowed to equilibrate for 10 min and this process was repeated ∼25 times. Upon exhaustive D_2_O exchange of the drop solution, crystals were mounted in a thin-walled, 2 mm diameter quartz capillary (Hampton Research) with a plug of deuterated reservoir and sealed with wax. The large DHP-B crystals used for neutron diffraction exhibited a different morphology to the smaller sized crystals that were previously used in X-ray diffraction experiments [Figs. 1 ▸(*a*) and 1 ▸(*d*)]. Specifically, they had a pyramidal or cubic morphology and did not resemble the trifold shape observed for smaller DHP-B crystals (McCombs, Moreno-Chicano *et al.*, 2017 ▸). The perdeuterated crystals did not grow as large as the protiated crystals, yet were still able to reach a sufficient volume for neutron diffraction [>0.1 mm^3^; Figs. 1 ▸(*a*) and 1 ▸(*b*)].

#### 2.1.5. Micro-crystallization and sample preparation

For micro-crystallization experiments (SSX, SFX and SLX), ferric DHP-B was recombinantly expressed and purified as described previously (McCombs *et al.*, 2016 ▸). In the case of oxyferrous DHP-B, the purification protocol was varied. After cell lysis, DHP-B was typically present in a mixture of oxyferrous and ferric forms. To isolate the oxyferrous form, the lysate was subjected to a salt cut up to 55% ammonium sulfate (de Serrano *et al.*, 2007 ▸), followed by centrifugation (18 000 rev min^−1^, 20 min). The supernatant was applied onto a phenyl Sepharose hydrophobic column (GE Healthcare) with a linear gradient from 1.5 to 0.0 *M* ammonium sulfate in 20 m*M* HEPES pH 7.5 buffer. The oxyferrous species eluted first. The oxyferrous DHP-B sample was further purified using size-exclusion chromatography (G-75, GE Healthcare) and its identity was validated by UV–Vis spectroscopy. In all cases, DHP-B microcrystals were grown in batch as reported previously (Moreno-Chicano *et al.*, 2019 ▸). For the SFX and SSX experiments, crystals were loaded onto silicon fixed-target chips in a humidity-controlled enclosure as described previously (Ebrahim, Appleby *et al.*, 2019 ▸). For the SLX data, 40–80 µl of the microcrystal suspension was loaded into each novel design chip (Supplementary Fig. S2; Doak *et al.*, 2018 ▸), with excess liquid manually blotted from the opposite chip face. All loaded chips were enclosed between two Mylar windows to maintain crystal hydration prior to mounting on the beamline.

#### 2.1.6. Neutron crystallographic data collection and reduction

RT neutron diffraction data were collected on the IMAGINE diffractometer at the High Flux Isotope Reactor (HFIR) at Oak Ridge National Laboratory (Meilleur *et al.*, 2013 ▸). From a 0.3 mm^3^ ferric perdeuterated DHP-B crystal, 20 frames of quasi-Laue data were collected utilizing a bandpass of 2.8–4.6 Å with 20 h exposure per frame. Each image was indexed and integrated using *LAUEGEN* (Campbell *et al.*, 1998 ▸). Wavelength normalization was performed with *LSCALE* (Arzt *et al.*, 1999 ▸) and the data were then scaled and merged with *SCALA* (Evans, 2006 ▸). The data extended to 1.95 Å resolution but were later truncated to 2.05 Å for structure refinement. RT X-ray diffraction data (data set NX-Xray) were subsequently collected from the same crystal using an in-house Rigaku MicroMax-007 HF rotating-anode generator (λ = 1.54 Å) equipped with an R-AXIS IV^++^ detector and consisted of 120 frames of 30 s exposure each. The frames were indexed and scaled using *HKL*-3000 (Minor *et al.*, 2006 ▸). Molecular replacement was performed with *Phaser-MR* in *Phenix* (Liebschner *et al.*, 2019 ▸) using PDB entry 3ixf (de Serrano *et al.*, 2010 ▸) as the search model. Model building and manual placement of waters utilized *Coot* (Emsley *et al.*, 2010 ▸) and refinement was carried out using *phenix.refine*. Upon the removal of waters and exogenous ligands, this RT X-ray structure (NX-Xray) served as the starting model for initial rigid-body refinement of the neutron structure against the neutron data alone. As the NX data were collected from a ferric DHP-B crystal, and the NX-Xray data were from an X-ray photoreduced form, the NX-Xray structure could not be used for joint refinement of the neutron structure.

D atoms were added to the starting model, with partial occupancy of hydrogen and deuterium at the exchangeable sites. Model building, manual placement of waters and refinement of the neutron structure were accomplished using the same method and programs as utilized for the X-ray structure. The X-ray and neutron structures were refined to resolutions of 1.75 and 2.05 Å, respectively.

The oxyferrous DHP-B neutron and X-ray diffraction data were collected and processed in the same manner using a 0.2 mm^3^ nonperdeuterated crystal. The nuclear density maps of oxyferrous DHP-B lacked definition in many hydrogen-containing regions due to the negative scattering length of H atoms. However, the X-ray data were again refined to give an oxyferrous DHP-B structure, allowing joint refinement using both neutron and X-ray scattering data. These complementary density maps allowed the structure to be clearly resolved. The oxyferrous X-ray and neutron data extended to resolutions of 1.95 and 2.20 Å, respectively, and the joint neutron/X-ray structure was refined to a resolution of 2.20 Å. The oxyferrous joint neutron/X-ray structure possessed a water molecule in the distal heme cavity that interacted with the O_2_ molecule bound to the iron.

### 2.2. SSX, SFX and SLX data collection from microcrystals and data reduction

#### 2.2.1. SSX data

SSX data were measured on beamline I24 at Diamond Light Source using silicon fixed-target chips as described previously (Owen *et al.*, 2017 ▸). Each exposure was of 20 ms, using an 8 × 8 µm beam at 12.8 keV, and diffraction data were measured using a PILATUS3 6M detector. The data-collection time for a full chip was ∼12 min. Data were processed using with *dials.stills_process* (Winter *et al.*, 2018 ▸) and merged using *Prime* (Lyubimov *et al.*, 2016 ▸). A total of 8131 indexed diffraction patterns produced a data set to a resolution of 1.45 Å. The total dose for the data set was estimated to be 82 kGy (Bury *et al.*, 2018 ▸)

#### 2.2.2. SFX data

SFX diffraction data were collected on beamline BL2 (EH3) at the SPring-8 Ångstrom Free-Electron Laser (SACLA) using the same sample stages and chip system as used for the SSX data (Ebrahim, Appleby *et al.*, 2019 ▸). The sample stages were mounted in a helium chamber to reduce air scatter. The X-ray pulse length was 10 fs, with an energy of 10.0 keV, a beam size of 1.25 × 1.34 µm and a repetition rate of 30 Hz. SACLA was in SASE mode with an X-ray bandwidth (FWHM) of ∼70 eV. Diffraction data were measured using the MPCCD detector (Kameshima *et al.*, 2014 ▸). Initial hit identification at the beamline used *Cheetah* (Barty *et al.*, 2014 ▸), while peak-finding, integration and merging were carried out using *CrystFEL* (White *et al.*, 2016 ▸). Data resolution was assessed based on outer shell correlation coefficient (CC) and *R*_split_ (White *et al.*, 2013 ▸) values together with the outcome of structure refinement (Table 1 ▸). SFX data for the oxyferrous form were also measured at BL2 (EH3) using a modified experimental setup without a helium enclosure. The X-ray energy was 11 keV, with a 30 Hz repeat rate and a 10 fs pulse duration.

#### 2.2.3. SLX data

SLX data were measured on beamline BioCARS 14-ID-B at the Advanced Photon Source (APS), Chicago, USA operating in 24-bunch mode using a 15 keV beam with a bandpass Δ*E*/*E* of ∼5% (FWHM) and a repeat rate of 2 Hz. Data were collected using a novel crystallo­graphic chip that avoids sample desiccation by encapsulating the chip in a sealed environment saturated with a desired hydrating solution (Supplementary Fig. S2; Doak *et al.*, 2018 ▸). An array of microscopic holes, etched through the Cyclotene film, allows crystal solution to be applied to one side of a window and then blotted carefully from the other side, with the intent of drawing crystals into the holes to accommodate stepwise raster scanning of the X-ray beam, hole by hole, across the chip. Since crystal solutions invariably contain a broad distributions of crystal sizes, the chips were not etched simply with holes of identical size but rather with a dual size distribution of small holes interspersed equally with large ones, as seen in Supplementary Fig. S3. Further descriptions of this novel fixed-target system and its development can be found in the supporting information.

Two exposure modes were used with 24 or 11 pulses of 100 ps each, with a total train duration of 3.6 or 1.6 µs, respectively, for a combined final data set. Each pulse contained 7.5 × 10^9^ photons. In order to maximize the resolution, the DHP-B microcrystals used for SLX were larger in size than those used for SFX and SSX, ranging between 40 and 50 µm. The average dose per crystal was estimated to be 21.8 kGy with *RADDOSE*-3*D* (Bury *et al.*, 2018 ▸), which allows pink-beam specifications. Images were recorded with a Rayonix MX340-HS detector. The BioCARS *pyPrecognition* Python script was used to find ‘hits’: diffraction patterns containing a specified number of spots above the background threshold level. Indexing of ‘hits’ was performed by the *Precognition* software. Indexed images were checked both automatically and visually to eliminate multiple diffraction patterns. Selected images containing single diffraction patterns were further processed by the *Precognition*/*Epinorm* software package to a resolution of 2.0 Å. Data were converted to mtz format in *CCP*4 prior to refinement in *Phenix*.

#### 2.2.4. SSX, SFX and SLX structure refinement and valid­ation

Structures were solved by molecular replacement using PDB entry 3ixf, a 1.58 Å resolution structure of dehalo­peroxidase B (de Serrano *et al.*, 2010 ▸), as a search model. Refinement was carried out in *REFMAC*5 (Murshudov *et al.*, 2011 ▸) via the *CCP*4*i*2 interface (Potterton *et al.*, 2018 ▸) and subsequently in *phenix.refine* (Adams *et al.*, 2013 ▸) with torsion-angle simulated annealing used in the early stages of refinement. Manual rebuilding was performed in *Coot* (Emsley *et al.*, 2010 ▸). Structures were validated using *QC-Check*, *MolProbity* (Williams *et al.*, 2018 ▸) and tools within *Phenix* (Liebschner *et al.*, 2019 ▸) and *Coot*. Superposition of structures was performed in *GESAMT* (Krissinel, 2012 ▸) or *Coot*. Images were prepared in *CCP*4*MG* (McNicholas *et al.*, 2011 ▸) and *PyMOL* (Schrödinger). Structures were deposited in the RCSB Protein Data Bank with the accession codes given in Tables 2 ▸ and 3 ▸. The oxyferrous DHP-B structure exhibited a significant level of twinning, and this was accounted for using twin refinement in *REFMAC*5. As a consequence, this structure was only refined in *REFMAC*5.

## 3. Results

### 3.1. Diffraction data quality and overall properties of the structures

Data were measured either from a single large crystal of volume 0.3 mm^3^ (NX, NX-Xray) or from batch-grown microcrystals of typical dimensions 15–25 µm (SFX) or 30–50 µm (SSX, SLX), all at RT (Tables 2 ▸ and 3 ▸). Sample-mounting/delivery systems are summarized in Supplementary Figs. S1–S3. As these are the first RT DHP-B structures, we have also provided a comparison with a previous structure of the enzyme determined under cryogenic conditions (PDB entry 3ixf). A comparison of the data-collection and processing statistics for all data sets and experimental methods is given in Tables 2 ▸, 3 ▸ and 4 ▸. The resolutions of the structures determined were between 1.45 and 2.05 Å. The cryogenic structure determined using synchrotron radiation (PDB entry 3ixf, resolution 1.58 Å) shows that DHP-B exists as a dimer in the asymmetric unit, which is also true for all of the structures that we describe here (Supplementary Fig. S4). The unit-cell dimensions were variable (by ∼1 Å in *a*, *b* and *c*; Table 2 ▸, Supplementary Fig. S5), but no specific trend was observed.

### 3.2. Neutron room-temperature crystal structure

Neutron diffraction data were collected from a perdeuterated ferric DHP-B crystal of ∼0.3 mm^3^ in volume (Fig. 1 ▸); data-collection and refinement statistics are provided in Tables 2 ▸, 3 ▸ and 4 ▸. Perdeuteration of DHP-B resulted in continuous nuclear density maps lacking the cancellation that can result from the negative neutron scattering length of hydrogen. There is clear density for the location of D atoms. An absence of nuclear density is noted for the hydroxyl group of some threonine, serine and tyrosine residues, which is likely to be due to incomplete D/H exchange. Rotational freedom or partial deuterium exchange at these positions are likely causes. However, the majority of exchangeable H atoms are visible in the NX maps, suggesting that they were successfully replaced with deuterium, in particular the proximal His89 and catalytically relevant distal His55 residues at the active site. Following the completion of neutron data collection, RT X-ray diffraction data were collected from the same crystal using an in-house source (NX-Xray).

**Figure 2:**
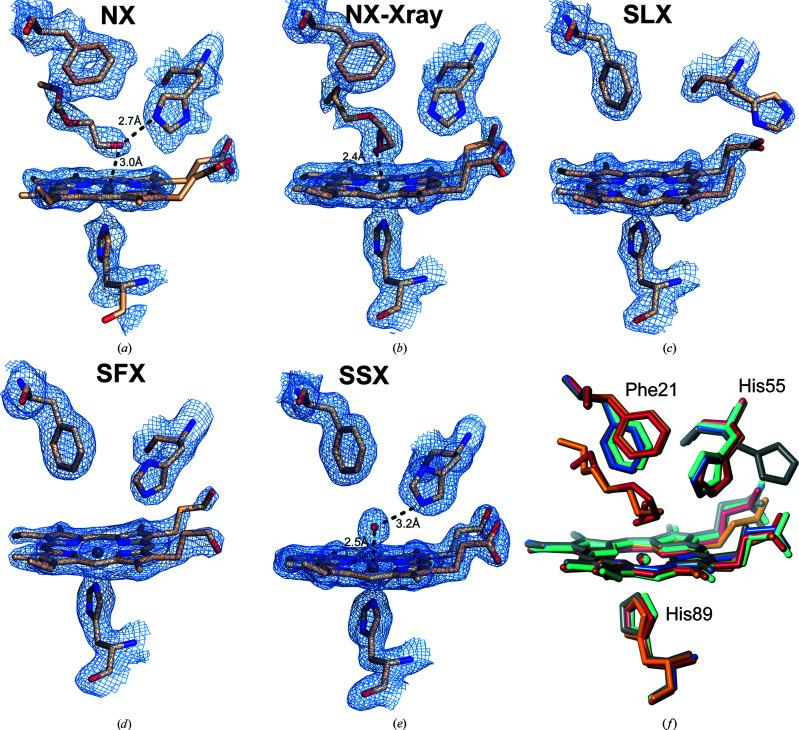
Heme sites with 2*F*_o_ − *F*_c_ electron or nuclear density maps contoured at 1σ for (*a*) the neutron structure and (*b*) the X-ray structure from the same crystal after the neutron structure had been obtained. Note the presence of a PEG molecule at the heme distal site in both cases. (*c*) Serial Laue structure showing a 5c site. (*d*) XFEL structure of DHP-B protomer *B*. (*e*) The synchrotron serial crystallography (SSX) structure. Note the distal water molecule coordinating the heme iron at the axial position. (*f*) Superposition of the nonhemichrome heme site for the different structures in this work. NX structure, orange; NX-Xray structure, red; SFX structure, blue; SSX structure, turquoise; SLX structure, grey.

Protomer *A* in the NX structure shows an MPEG molecule from the crystallization medium coordinated to the heme Fe at a distance of 3 Å [Fig. 2 ▸(*a*)]; UV–visible spectroscopic data supporting the binding of MPEG to ferric DHP-B in solution are provided in Supplementary Fig. S6. His55 is neutral and is positioned interior to the distal cavity, stabilized by a hydrogen-bond interaction with the MPEG at 2.74 Å. The MPEG extends into the distal pocket towards the heme α–δ edge, flanked by nonpolar (Val69) and aromatic (Phe21 and Phe60) residues. The latter residue is observed in a conformation with the phenyl ring displaced towards the back of the pocket from its typical position in DHP structures with no MPEG bound (Moreno-Chicano *et al.*, 2019 ▸), suggesting that it has moved to accommodate the binding of MPEG. Tyr38 is also positioned into the heme cavity and is stabilized through a stacking interaction with Phe35 (Supplementary Fig. S6). In the proximal region, His89 N^ɛ^ is positioned 2.41 Å from the heme Fe. As previously assigned from previous X-ray crystal structures, hydrogen bonding between His89 N^δ^ (deuterated) and the carbonyl of Leu83 was verified in the neutron structure at a distance of 2.56 Å. This interaction provides DHP with the proximal charge relay that is traditionally required for heme activation, as DHP lacks the canonical Asp–His–Fe proximal catalytic triad that is found in most peroxidases.

**Figure 3:**
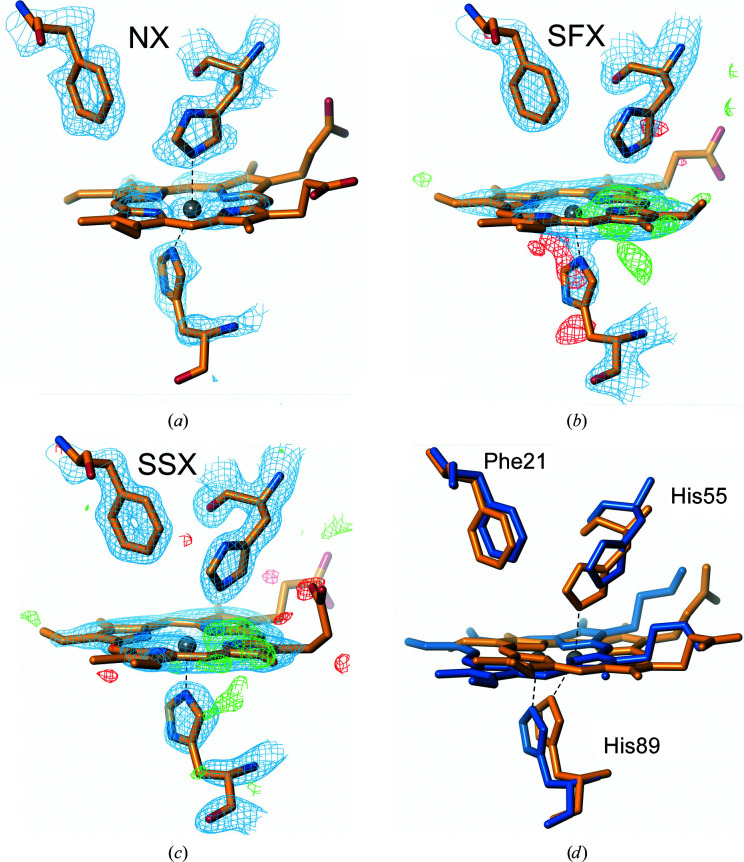
Hemichrome sites shown for different DHP-B structures. (*a*) Neutron crystallography structure (NX). (*b*) Serial femtosecond crystallography structure (SFX). (*c*) Serial synchrotron structure (SSX). (*d*) Superposition of the fully occupied hemichrome found in the neutron structure (orange) with the nonhemichrome heme site from the SFX structure (blue). Note the heme shift towards the enzyme surface upon the formation of the hemichrome, as well as the conformational changes of the ligating His residues. Relevant coordination bonds between the heme iron and the proximal and distal histidine ligands, including those involved in the hemichrome species, are shown as black dashed lines. All electron-density (2*F*_o_ – *F*_c_) maps are shown as a blue mesh and contoured at 1σ. Difference density (*F*_o_ – *F*_c_) maps are shown as a green or red mesh for positive or negative differences, respectively, and are contoured at 3σ.

In protomer *B*, in addition to the proximal His89 ligand (2.54 Å), the distal His55 (2.48 Å) is coordinated directly to the iron [Fig. 3 ▸(*a*)], yielding a bis-His hemichrome species in which the heme is positioned slightly out of the cavity and Tyr38 is positioned into the heme cavity. This hemichrome structure in one protomer is also found with partial occupancy in X-ray structures determined at 100 K and for the RT structures derived from microcrystals and large single crystals (see below). In the DHP hemichrome the heme is slightly extruded from the distal pocket [Fig. 3 ▸(*d*)].

Protomer *A* in the NX-Xray and NX structures shows an MPEG molecule and an O_2_ molecule, each with partial occupancy, coordinated to the heme Fe [Fig. 2 ▸(*b*)]. The Fe distance to O-MPEG in the NX-Xray structure is 2.23 Å, which is much shorter than in the NX structure (3.04 Å). During RT data collection, we assume that the ferric heme centre of protomer *A* was rapidly photoreduced to the ferrous state due to the ionizing properties of the X-ray beam, which is consistent with extensive studies on heme proteins (see, for example, Beitlich *et al.*, 2007 ▸) and specifically of DHP (McCombs, Moreno-Chicano *et al.*, 2017 ▸). This reduction could lead to displacement of the MPEG molecule as DHP forms the oxyferrous state. Consistent with this, an O_2_ molecule with a partial occupancy of 0.32 is modelled with an Fe—O distance of 2.41 Å [Fig. 2 ▸(*b*)].

Both the distal histidine (His55) and proximal histidine (His89) in the neutron structures are shown not to be charged. The hydrogen bond between His89 N^δ^-D and the carbonyl O atom of the Leu83 backbone is visualized, as previously inferred in X-ray structural data. This interaction is mechanistically important in the ability of DHP-B to perform both oxygen transport and oxidative functions. In the hemichrome species (protomer *B*), His55 is hydrogen-bonded to a water molecule in the distal pocket of the oxyferrous structure, strongly suggesting that this is a stable interaction in lieu of the lack of observable D atoms. No distal water is observed in the ferric hemichrome species. In protomer *A*, the rotation of His55 differs between the ferric and oxyferrous neutron structures. In the ferric structure N^δ^ is protonated and interacts with the propionate arms, while N^ɛ^ positions itself as a hydrogen-bond acceptor for the PEG ligand. However, in the oxyferrous structure N^δ^ is protonated and is rotated toward the O_2_ ligand, positioning itself as a hydrogen-bond donor (see Supplementary Table S3 for the extent of deuterium exchange in the active site).

### 3.3. Serial crystallography structures of DHP-B

The damage-free SFX structure was determined at the SACLA XFEL to a resolution of 1.85 Å. The heme environment was well defined in both protomers of the SFX structure. In protomer *B* His55 was in a conformation swung into the distal pocket [Fig. 2 ▸(*d*)]. In contrast, in a previous 100 K synchrotron-radiation structure obtained from initially ferric crystals (PDB entry 3ixf), His55 was swung out of the pocket. Another feature of this site is the additional difference electron density found in the axial position above the heme iron, which suggests the partial occupancy of a distal water coordinating the iron in the ferric state. This observation is supported by resonance Raman spectroscopy data of ferric DHP in solution. Raman spectroscopy indicates the existence of an equilibrium between a pentacoordinate and a hexacoordinate (water) high-spin species of ferric DHP-B (D’Antonio *et al.*, 2010 ▸) that would only be consistent with partial occupancy of this site. Modelling of a water molecule in this position with an occupancy of 0.3 produces a very weak 2*F*_o_ − *F*_c_ electron-density peak, with an approximate Fe—O distance of 2.5 Å. Assignment of this distance is rendered difficult by the iron being displaced away from the water position towards the proximal His in the majority of DHP-B molecules where water is not present.

In protomer *A*, where a partially occupied hemichrome species was observed in previous structures at 100 K (data not shown), a double conformation of the heme can also be appreciated, although not as clearly as in the 100 K data, likely due to the lower resolution. However, a clear negative *F*_o_ − *F*_c_ difference density peak [Fig. 3 ▸(*b*)] can be seen for the main heme iron and additional positive density is observed in the second site which is coordinated by a His55 conformation closer to the heme plane than in protomer *B*. An extra positive *F*_o_ − *F*_c_ difference density peak is also observed in the proximal site just beneath the putative location of the second iron site, suggesting an alternative conformation of the main proximal histidine to form the hexacoordinated species characteristic of the hemichrome form.

A structure at 1.45 Å resolution was determined by SSX on beamline I24 at Diamond Light Source. The overall fold of each protomer was identical to that obtained by SFX, with r.m.s.d.s of 0.46 Å for protomer *A* and 0.44 Å for protomer *B*. In protomer *A*, a partially occupied hemichrome species was observed in the same manner as for the SFX structure [Fig. 3 ▸(*c*)], although in this case with higher occupancy. In contrast, at the heme site of protomer *B* a strong positive difference electron-density feature was observed that could be modelled as a water molecule with an Fe—O distance of 2.6 Å stabilized by a hydrogen bond to His55 at 3.2 Å [Fig. 2 ▸(*e*)]. Alternatively, the feature could be modelled as a dioxygen molecule, which would be consistent with either autoreduction to the oxyferrous state *in crystallo* or to the effects of radiation damage. Nevertheless, formation of the oxyferrous species from an irradiated ferric DHP-B crystal was not observed in a previous dose-series experiment at much higher doses (a set of eight sequential X-ray crystal structures from the same exposed region, unpublished data; the highest dose was 3.6 MGy compared with 82 kGy for SSX) using crystals at 100 K.

The serial Laue crystallography (SLX) structure was determined to 2.0 Å resolution on the BioCARS 14-ID-B beamline at the Advanced Photon Source utilizing a novel chip (see Section 2 and supporting information). In this structure, the heme iron is pentacoordinate in both protomers, with no evidence of a distal water or dioxygen ligand. There is no electron density to indicate a hemichrome species in protomer *A* in this case. Notably, in both protomers the distal His55 residue is swung away from the heme iron and towards the protein surface [Fig. 2 ▸(*c*)]. A water molecule has been modelled in the distal pocket of protomer *B*. Due to its position, this density could also arise from the alternate swung-in conformer of His55, particularly since a water molecule at this position would not be stabilized by hydrogen bonding to any protein residue or heme propionate.

### 3.4. Characterization of oxyferrous structures

In two cases (SFX and NX), we obtained data for an iron(II) form of DHP-B with a dioxygen molecule bound to the heme iron. The SFX structure again reveals a marked asymmetry between protomers: in protomer *A* no distal ligand was observed, while in protomer *B* a clear density feature was evident at the distal coordination position (Supplementary Fig. S8). This was successfully modelled as an oxygen molecule consistent with an oxyferrous form of DHP-B. The Fe—O distance was 2.0 Å and the Fe—O—O angle was 136°. Intriguingly, no evidence of hemichrome formation was apparent in this structure. In the oxyferrous structure obtained by NX we also observe an oxygen molecule bound to the heme in protomer *A*, with an Fe—O bond distance of 2.55 Å and a closer-to-linear Fe—O—O angle of 164° (Supplementary Fig. S8). A comparison of data from SFX and NX is given in Supplementary Fig. S8.

## 4. Discussion

In this wide-ranging study, we have determined the first RT crystal structures of DHP-B using several complementary crystallographic diffraction methods that allow atomic-level structure determination at room temperature. The different approaches produced structures with resolutions in the range 2.05–1.45 Å, allowing an effective comparison between them to be made. Such comparisons must be made with the caveat that the ability to model and resolve structural features changes significantly over this resolution range, with multiple conformations of side chains and cofactors, ligand identity and pose, and lower occupancy states being substantially easier to identify at resolutions of ∼1.5 Å or better. The methodo­logical approaches used vary substantially in the number and size of crystals required and the form of the data produced; issues that we discuss here in detail.

### 4.1. Comparison of overall structures

The overall crystal structures of DHP-B obtained by SFX, SSX and SLX using microcrystals are highly similar and may be superposed with r.m.s.d. values in the range 0.29–0.31 Å (all protein atoms). The NX and NX-Xray structures can be superposed with the SFX structure with increased r.m.s.d. values of 1.28 and 1.31 Å (all protein atoms), respectively. This difference can be rationalized by the fact that these structures were obtained from crystals grown under different crystallization conditions and also represent a ligand-bound state (MPEG molecule). A superposition of the different structures is shown in Supplementary Fig. S9 and a superposition of the heme sites can be seen in Fig. 2( ▸ *f*). Pairwise r.m.s.d. values are also given in Supplementary Fig. S10. Multiple previous structures of DHP-B have been determined under cryogenic conditions (de Serrano *et al.*, 2010 ▸; McCombs, Moreno-Chicano *et al.*, 20.17 ▸; Carey *et al.*, 2018 ▸). Despite the *B* factors being smaller for structures determined at 100 K compared with those reported at RT here, the overall structural features are maintained. The temperature-dependent difference in *B* factors is expected given the difference in energy landscape and the greater conformational variability at room temperature (Russi *et al.*, 2017 ▸).

### 4.2. Effects of crystal size and method on structure and data quality

Our data allow the comparison of RT structures obtained from large single crystals and microcrystals, all in the same space group and with comparable unit cells, and hence without a confounding influence from differing crystal-packing effects or from major differences in crystallization conditions and crystal composition. Refinement *R* factors, resolution limits and geometrical parameters are given in Tables 2 ▸, 3 ▸ and 4 ▸. Real-space *B* factors varied between the structures, with the mean *B* factors for all macromolecular atoms being 44.6 Å^2^ (SFX), 17.6 Å^2^ (NX), 25.4 Å^2^ (SSX), 28.0 Å^2^ (NX-Xray) and 18.3 Å^2^ (SLX). Related to the *B* factor and the quality of the experimental data is the estimated coordinate error assessed using the diffraction precision indicator (DPI; Kumar *et al.*, 2015 ▸). As expected, the DPI is higher for all RT structures in comparison with structures determined at 100 K, due to the higher level of thermal motion represented in part by the *B* factor (Table 4 ▸).

### 4.3. Comparison of damage-free SFX and neutron structures

Two structures, NX and SFX, were obtained free of the effects of radiation damage, enabled for SFX by the short (10 fs) X-ray pulse length of the XFEL radiation. SFX and NX provide complementary structural information. Both the SFX and NX structures were determined to high resolution. The structures superimposed with r.m.s.d. values of 0.67 Å (protomer *A*) and 0.64 Å (protomer *B*) (Supplementary Fig. S9). In both structures His55 is oriented towards the iron, with no evidence of a ‘swung-out’ conformation of this residue. In our SLX data, difference density indicates a weak area of positive electron density suggesting a dual conformation of His55 (data not shown). Modelling of a dual conformation for this residue did not prove stable in refinement and so only the primary conformation was included in the final model. The position of the distal pocket residues Phe21 and Phe60 in the neutron structure is close to that observed in ligand-bound structures of DHP and is consistent with the presence of a PEG molecule in the distal pocket, and differs from that in the ferric SFX structure. Both the SFX and neutron approaches were effective as a way of obtaining damage-free structures and may be seen as complementary, with higher resolution from SFX but with light atoms (H/D) being identified from the neutron data. The origin of the observed structural differences between these methods may be explained by the utilization of different crystallization conditions that were needed to obtain the desired crystal characteristics in each case. Interestingly, the utilization of different PEGs (MPEG 2000 for the NX structure and PEG 4000 in the case of SFX) yielded two different ‘states’ of the hemichrome (fully and partly occupied), suggesting its involvement in the formation of this species. Hemichrome species have been well characterized in hemoglobins (Riccio *et al.*, 2002 ▸), occurring either reversibly or nonreversibly, and in some but not all cases are linked to partial denaturation of the protein, for example as caused by exposure to agents such as polyethylene glycol. Reversible hemichromes have been suggested to be a normal conformational substate of hemoglobin. Other speculative possibilities include the long exposure to RT (the crystals were grown at 4°C) needed for NX data collection. In principle, structural variance could also arise between NX or NX-Xray and the other structures as a result of perdeuteration.

While all structures were determined using crystals grown from ferric DHP-B solution, there is considerable variation in the active-site structures. Factors that may contribute to this include the data-collection time and temperature, different crystallization conditions and perdeuteration, together with crystal size and variation in crystal age. For the SSX, SLX and NX-Xray structures site-specific radiation damage may also be a contributing factor.

A second pair of damage-free structures are those of oxyferrous DHP-B determined by NX and SFX. In the case of SFX, the crystals were grown from a DHP-B sample that was isolated in the oxyferrous form using column chromatography (see Section 2). For NX, the oxyferrous state arose from chemical reduction. Active sites are shown in Supplementary Fig. S8. Both NX and SFX were able to clearly distinguish between the ferric and oxyferrous heme-pocket structures. Unexpectedly, an oxygen molecule was only observed in one protomer of the SFX structure, despite the protein solution used in crystallization exhibiting a typical visible absorption spectrum for oxyferrous protein (data not shown; D’Antonio & Ghiladi, 2011 ▸), suggesting that there may be partial reoxidation or loss of the ligand.

### 4.4. Definition of the hemichrome species in room-temperature structures

In many structures of DHP there is evidence for the formation of a hexacoordinate ‘hemichrome’ structure in which the heme moiety is displaced significantly towards the enzyme surface from its position in the nonhemichrome form and, at the distal pocket, His55 becomes coordinated to the heme iron, resulting in a hexacoordinate heme (see, for example, McCombs, Moreno-Chicano *et al.*, 2017 ▸; Jiang *et al.*, 2013 ▸; Franzen *et al.*, 2012 ▸). This species has been speculated to be a candidate for the protective reversible hemichrome or ‘compound RH’ proposed from solution spectroscopic studies where DHP is exposed to H_2_O_2_ in the absence of substrate (Feducia *et al.*, 2009 ▸; Thompson *et al.*, 2010 ▸).

Previous suggestions for the origin of the hemichrome formed in crystals (in the absence of H_2_O_2_) were exposure to glycerol during cryoprotection or the effects of radiation damage. The data presented here rule out these possibilities as the crystals have not been exposed to glycerol and the neutron and SFX structures are damage-free. It is possible that this species is present in recombinant DHP-B prior to crystallization or alternatively that crystallization itself produces this change, for example by interactions with the polyethylene glycol precipitant. Interestingly, the observation of a hemichrome species in RT data sets argues against the possibility that this is caused by exposure to dehydrating cryoprotectants such as glycerol.

In the present study the hemichrome species provides a useful common point for comparison between structures, since it is present to some extent in most of the structures (Fig. 3 ▸). Notably, in the NX and X-ray structures protomer *B* of the dimer appears to be entirely in the hemichrome form. In contrast, in protomer *B* of the SFX structure the heme is predominantly in its nonhemichrome (5c) form, but a large peak in the *F*_o_ − *F*_c_ difference map indicates a second position of the Fe atom. In the SSX structure (protomer *A*) the His55 side chain is again oriented towards the heme, with a difference map peak for a second Fe position, and the ligating His89 can be modelled in two conformations. In the SLX structure there is no evidence of hemichrome formation.

### 4.5. Radiation damage in room-temperature DHP-B structures

The SSX, SLX and NX-Xray structures incurred X-ray doses ranging from 21.8 to 83 kGy. While these are very low doses, small enough to avoid typical site-specific damage to bound ligands or proteins such as decarboxylation of side chains or reduction of disulfide bonds (Ebrahim, Moreno-Chicano *et al.*, 2019 ▸), we expect the photoreduction of the heme iron to occur after just a few kGy (Kekilli *et al.*, 2017 ▸; McCombs, Moreno-Chicano *et al.*, 2017 ▸). The observation of conformational change of His55 in the ‘swung-out’ position, in contrast to its ‘swung-in’ conformation in the damage-free structures (SFX and NX), implies another site-specific radiation-damage effect. Conformational changes of side chains at the active site upon X-ray-driven reduction have been reported for heme proteins by Kekilli *et al.* (2014 ▸, 2017 ▸) and are more apparent at RT than at 100 K (Atakisi *et al.*, 2019 ▸; Gotthard *et al.*, 2019 ▸).

### 4.6. General comments on room-temperature methods for the study of DHP and other heme proteins

We have described the very different sample requirements, data-collection times, crystal sizes and X-ray source regimes for the methods used in this study (Table 2 ▸). Choices of which method to use for a particular study may be influenced by these factors, for example the ability to produce large quantities of protein and whether suitable microcrystals may be generated. Similarly, the availability of methods varies, with XFEL-based methods and neutron diffraction being particularly challenging to access. The other side of the coin is whether particular methods offer unique advantages to answer particular biological questions for the protein of interest. For example, neutron diffraction can answer very specific questions on the protonation states of key residues related to enzyme mechanism in a manner that the other methodologies cannot. If the heme-protein states of interest are particularly sensitive to X-ray radiation damage, as is the case for iron(IV) states, then SFX or very low dose SSX may be well suited to capture intact or near-intact states, respectively. In contrast, radiation damage may be a lesser concern where the aim is to capture a reduced, iron(II) state which may not be significantly altered at the doses typically used in X-ray diffraction experiments.

More generally, there may be a premium on high resolution to be able to identify the precise locations of mechanistically important residues and to identify multiple conformations and partial states that may be more evident at room temperature than under cryogenic conditions. Our study cannot provide clear guidance for which method will produce the highest resolution data, given that the experimental variables for all of the methods used could be modified to achieve this.

There are clear advantages in combining different room-temperature experimental methods to generate structures in that this allows the unique data available from neutron diffraction (*i.e.* protonation states) and the effects of radiation damage to be taken into account when interpreting structures and relating them to function.

In the work presented here, the ability to compare particular structural features using different methods was compromised to some extent by the underlying differences in structure in the crystals used for each. The protein fold was consistent between structures, while the coordination of the heme appeared to be partly variable, with a PEG molecule being present in the distal pockets of the large crystals grown for neutron diffraction. Similarly, serial crystallographic structures obtained by SFX and SSX differed in the presence of a water molecule at the distal face of the heme. The extent of hemichrome formation was also inconsistent, although the ability to distinguish this could be limited by differences in resolution.

## 5. Conclusions

The number of crystals, total quantity of protein and data-collection time required to determine each structure were very different between the different methods used. The total time required for the measurement of data sets varied greatly depending on the method used, although all were rather longer than that required for a typical single-crystal X-ray diffraction experiment at a synchrotron beamline. For serial crystallography experiments, the total times required to measure the data were 14 min (SSX), 70 min (SFX) and 78 min (SLX) (Table 2 ▸). Neutron diffraction experiments are inherently slower due to the low flux, and so some 400 h was required for data collection from a single crystal. The exposure time range for a single diffraction pattern was from 10 fs (SFX) to 20 h (NX). A major advantage of the Laue method is that far fewer (181) crystals were required for the Laue approach than for the other microcrystal-based approaches, with the drawback of more challenging data processing (Ren *et al.*, 1999 ▸; Gevorkov *et al.*, 2020 ▸). Currently, this is less automated in data collection and analysis but holds substantial promise for further development.

NX and SFX are complementary approaches to understand the structures of radiation-sensitive metalloproteins under non­cryogenic conditions, with the definition of heavier atoms being provided by SFX and H/D-atom positions uniquely being provided by NX. Comparable low-dose structures may be provided by SSX, using synchrotron beamlines where beamtime is more readily available and where high-quality structures may be obtained with modest radiation-damage effects. SLX is particularly valuable where limited numbers of microcrystals are available, requiring far fewer crystals than are needed for either SSX or SFX. In summary, we demonstrate that room-temperature NX, SFX, SLX and SSX can accurately and consistently define the active sites of DHP-B, with the observed differences only being due to the different crystals used for the respective methods. Neutron diffraction allowed us to define the positions of light atoms, while the different serial methods allowed high-resolution room-temperature structures to be determined, with different amounts of crystalline material being required. SFX and NX allowed structures to be determined free of the manifestations of radiation damage. SSX gave rise to the highest resolution serial structure, while SLX allowed structure determination using the fewest crystals of any serial method.

## 6. Related literature

The following reference is cited in the supporting information for this article: Chenprakhon *et al.* (2010 ▸).

## Supplementary Material

PDB reference: ferric DHP-B, neutron diffraction structure, 7jor

PDB reference: X-ray structure from crystal used for neutron diffraction, 7kfm

PDB reference: serial femtosecond crystallography structure, 7adf

PDB reference: serial synchrotron crystallography structure, 7acp

PDB reference: serial Laue crystallography structure, 7adq

PDB reference: oxyferrous DHP-B, joint X-ray/neutron structure, 7kcu

PDB reference: serial femtosecond crystallography structure, 7adx

Supplementary Tables and Figures. DOI: 10.1107/S2052252522006418/rs5001sup1.pdf

## Figures and Tables

**Table 1:** Nomenclature for the room-temperature crystal structures described in this study

NX	Structure obtained by neutron crystallography from a single ferric crystal
NX-Xray	X-ray crystal structure obtained from the same crystal as used for NX
SFX	Serial femtosecond crystallography structure obtained from microcrystals at the SACLA XFEL
SSX	Serial synchrotron crystallography structure obtained from microcrystals on beamline I24 at Diamond Light Source
SLX	Serial Laue crystallography structure obtained from microcrystals at the Advanced Photon Source BioCARS beamline
NX-Oxyf	Structure obtained by neutron crystallography from a single oxyferrous crystal
NX-Oxyf-Xray	X-ray crystal structure obtained from the same crystal as used for NX-Oxyf

**Table 2:** Data-collection and processing statistics for RT DHP-B crystal structures in space group *P*2_1_2_1_2_1_ Values in parentheses are for the outermost resolution shell. n.d., not determined.

	NX	NX-Xray	SFX	SSX	SLX
Source/beamline	HFIR/IMAGINE ORNL	Rigaku MicroMax-007	SACLA/BL2 (EH3)	Diamond/I24	APS/BioCARS
Data-collection time (min)	24000	60	42	14	78
Wavelength (Å)	2.8–4.6	1.54	1.13	0.969	0.827
Typical crystal volume (mm^3^)	0.2	0.2	1.5 × 10^−5^	6.25 × 10^−5^	7.5 × 10^−5^
Absorbed X-ray dose (kGy)	0	n.d.	0†	82	21.8
No. of images merged	20	90	10793	8181	181
*a*, *b*, *c* (Å)	60.8, 67.1, 69.0	60.8, 67.1, 69.0	61.3, 68.1, 68.3	61.2, 67.0, 68.9	60.8, 67.3, 67.5
Unit-cell volume (Å^3^)	273083	282398	285581	282518	276927
Resolution (Å)	17.48–2.20 (2.28–2.20)	48.13–1.95 (2.02–1.95)	48.2–1.85	68.9–1.45 (1.50–1.45)	47.7–2.00 (2.08–2.00)
No. of reflections	11267 (881)	21117 (2077)	25099 (1213)	50894	12928
*R*_split_ (%)	—	—	12.0 (60.0)	12.1 (65.8)	—
CC_1/2_	0.95 (0.72)	0.99 (0.94)	0.98 (0.50)	98.0 (53.3)	—
*R*_merge_ (%)	27.4 (41.2)	2.2 (13.5)	—	—	—
*R*_p.i.m._	10.6 (19.9)	2.2 (13.5)	—	—	—
*R*_merge_ on *F*	—	—	—	—	7.2
〈*I*/σ(*I*)〉	4.7 (2.2)	93.3 (8.5)	6.8	3.81 (0.74)	34.1 [*F*/sig*F*]
Multiplicity	5.5 (3.7)	2.0 (1.9)	322 (224)	104 (6.5)	7.55
Completeness (%)	78.0 (62.2)	99.5 (98.4)	100 (100)	100 (99.6)	67.8 (22.0)
Wilson *B* factor (Å^2^)	35.5	35.5	28.1	14.2	10.9

† The effective dose for the structure is quasi-zero due to the ‘diffraction before destruction’ principle associated with the short (10 fs) X-ray pulse.

**Table 3:** Refinement and validation statistics for RT DHP-B structures

Structure	NX	NX-Xray	SFX	SSX	SLX
No. of unique reflections	14124	28918	25055	50848	12928
*R*_work_	0.247	0.165	0.178	0.167	0.162
*R*_free_	0.289	0.194	0.213	0.209	0.215
R.m.s.d., bond lengths (Å)	0.017	0.007	0.004	0.003	0.003
R.m.s.d., bond angles (°)	1.62	0.88	0.80	0.50	0.44
Protein residues	274	274	274	274	274
Solvent molecules	46	122	89	173	144
Sulfates	0	2	2	2	2
Most favoured (%)	95.7	98.2	98.2	98.2	97.8
Overall coordinate DPI (Å)	0.365	0.095	0.128	0.081	0.180†
PDB code	7jor	7kfm	7adf	7acp	7adq

† ML-based ESU from *REFMAC*5. The online DPI server (Kumar *et al.*, 2015 ▸) requires a minimum completeness value to generate DPI values. The completeness did not allow a DPI to be calculated using the DPI server.

**Table 4:** Heme environment parameters for ferric DHP-B structures

State	Structure	Protomer	Resolution (Å)	Fe–His89 N^δ2^ (Å)	Fe–His55 N^ɛ2^ (Å)	Fe–ligand (Å)
Ferric	NX	*A*	2.05	2.26	5.32	3.21
		*B*		2.35	2.64	—
Oxyferrous†	NX-Xray	*A*	1.75	2.34	5.48	2.23/2.14‡
		*B*		2.39	2.58	—
Ferric	SFX	*A*	1.85	2.22	4.06	—
		*B*		2.17	4.94	—
Ferric	SSX	*A*	1.45	2.27	3.26	2.62
		*B*		2.15	5.01	—
Ferric	Laue	*A*	2.00	2.19	10.13	—
		*B*		2.23	10.14	—
Oxyferrous	SFX	*A*	1.85	1.94	4.13	—
		*B*		1.95	4.91	2.06
Oxyferrous	NX§	*A*	2.20	2.16	5.15	2.70
		*B*		2.37	3.09	—

† The crystal was originally ferric but was likely to have been reduced during X-ray data collection.

‡ The first number defines the distance to the MPEG oxygen and the second defines the distance to the heme-coordinated active-site O_2_.

§ Joint refinement of NX and X-ray data.
