# Supplementary material for: Complementarity of neutron, XFEL and synchrotron crystallography for defining the structures of metalloenzymes at room temperature
Source: IUCrJ. 2022 Jul 25;9(Pt 5):610–24. doi: 10.1107/S2052252522006418 (PMC9438502; doi:10.1107/S2052252522006418)
Supplement: Supplementary file 1 [file m-09-00610-sup1.pdf]

IUCrJ

Volume 9 (2022)

Supporting information for article:

Complementarity of neutron, XFEL and synchrotron crystallography for defining the structures of metalloenzymes at room temperature

Tadeo Moreno-Chicano, Leiah M. Carey, Danny Axford, John H. Beale, R. Bruce Doak, Helen M. E. Duyvesteyn, Ali Ebrahim, Robert W. Henning, Diana C. F. Monteiro, Dean A. Myles, Shigeki Owada, Darren A. Sherrell, Megan L. Straw, Vukica Šrajer, Hiroshi Sugimoto, Kensuke Tono, Takehiko Tosha, Ivo Tews, Martin Trebbin, Richard W. Strange, Kevin L. Weiss, Jonathan A. R. Worrall, Flora Meilleur, Robin L. Owen, Reza A. Ghiladi and Michael A. Hough

Table S1 Data collection and processing statistics for RT crystal structures of oxyferrous DHP-B in space group $P2_12_12_1$.

NX; neutron crystallography data from ORNL. NX-Xray; single crystal dataset obtained from the same crystal used for neutron diffraction; SFX, serial femtosecond crystallography at SACLA.

**Structure**	**NX**	**NX-Xray**	**SFX**
Source/Beamline	HFIR/Imagine ORNL	Rigaku MicroMax-007	SACLA/BL2 EH3
Data collection time (minutes)	24000	60	42
Wavelength (Å)	2.8 – 4.6	1.54	1.13
Typical crystal volume (mm^3)	0.2	0.2	$1.5x10^{-5}$
Effective absorbed X-ray dose (kGy)	0	n.d	0*
Number of crystals	1	1	10807
Number of images used	20	90	10793
Unit cell dimensions (Å)	60.8<br>67.1<br>69.0	60.8<br>67.1<br>69.0	61.3<br>68.1<br>68.3
Resolution (Å)	17.48-2.20<br>(2.28-2.20)	48.13-1.95$^{\$}$<br>(2.02-1.95)	48.2-1.85
Number of reflections	11267 (881)	21117 (2077)	25099 (1213)
R_{split}	-	-	12.0 (60.0)
$CC_{1/2}$	0.95 (0.72)	0.99 (0.94)	0.98 (0.50)
R_{merge}	27.4 (41.2)	2.2 (13.5)	-
R_{pim}	10.6 (19.9)	2.2 (13.5)	-
I/σ(I)	4.7 (2.2)	93.3 (8.5)	6.8
Multiplicity	5.5 (3.7)	2.0 (1.9)	322 (224)
Completeness (%)	78.0 (62.2)	99.5 (98.4)	100 (100)
Wilson B-factor ($Å^2$)	35.5	35.5	28.1

*Effective dose is zero due to the 'diffraction before destruction' principle associated with the short (10 fs) X-ray pulse. $^{\$}$Resolution and binning for data collection step. During refinement, the resolution was dropped to 2.2 Å to correlate with the neutron data.

Table S2 Refinement and Validation Statistics for RT oxyferrous DHP-B structures.

**Structure**	**NX**	**NX-Xray**	**SFX**
Number of reflections	11263	14803	23811
R_{work}	0.249	0.155	0.165
R_{free}	0.305	0.212	0.192
RMSD bond lengths (Å)	0.019		0.009
RMSD bond angles (°)	1.9		1.6
Protein residues	274		274
Water/D_2O molecules	106		85
Sulfates	2		2
Most favoured (%)	97		98.2
Overall co-ordinate DPI (Å)	0.622		0.125
PDB accession code	7KCU	7KCU	7ADX

Table S3 Extent of deuterium exchange on active site residues observed in neutron structures (%).

	Ferric neutron structure			
	**Protomer A**		**Protomer B**	
	N-H backbone	sidechain	N-H backbone	sidechain
H89	60	Nδ 69	65	Nδ 55
H55	51	Nδ 89	53	Nδ 64
T56	44	45	26	99
Y38	96	O-D 0	93	O-D 86
PEG terminal O-D 64			---	---

	Oxyferrous neutron structure			
	**Protomer A**		**Protomer B**	
	N-H backbone	sidechain	N-H backbone	sidechain
H89	99	Nδ 100	57	Nδ 74
H55	100	Nε 48	75	Nδ 36
T56	71	53	0	0
Y38	100	O-D 86	100	O-D 100
distal cavity water 0			distal cavity water 0	

Considering the distal cavity is predominantly hydrophobic, there are very few residues with H-D exchangeable sites on the sidechain. In both structures, Protomer A presents ligands in the distal pocket while Protomer B in both structures exists as a hemichrome species. No exchange was observed for the heme propionate arms, showing they are deprotonated as expected.

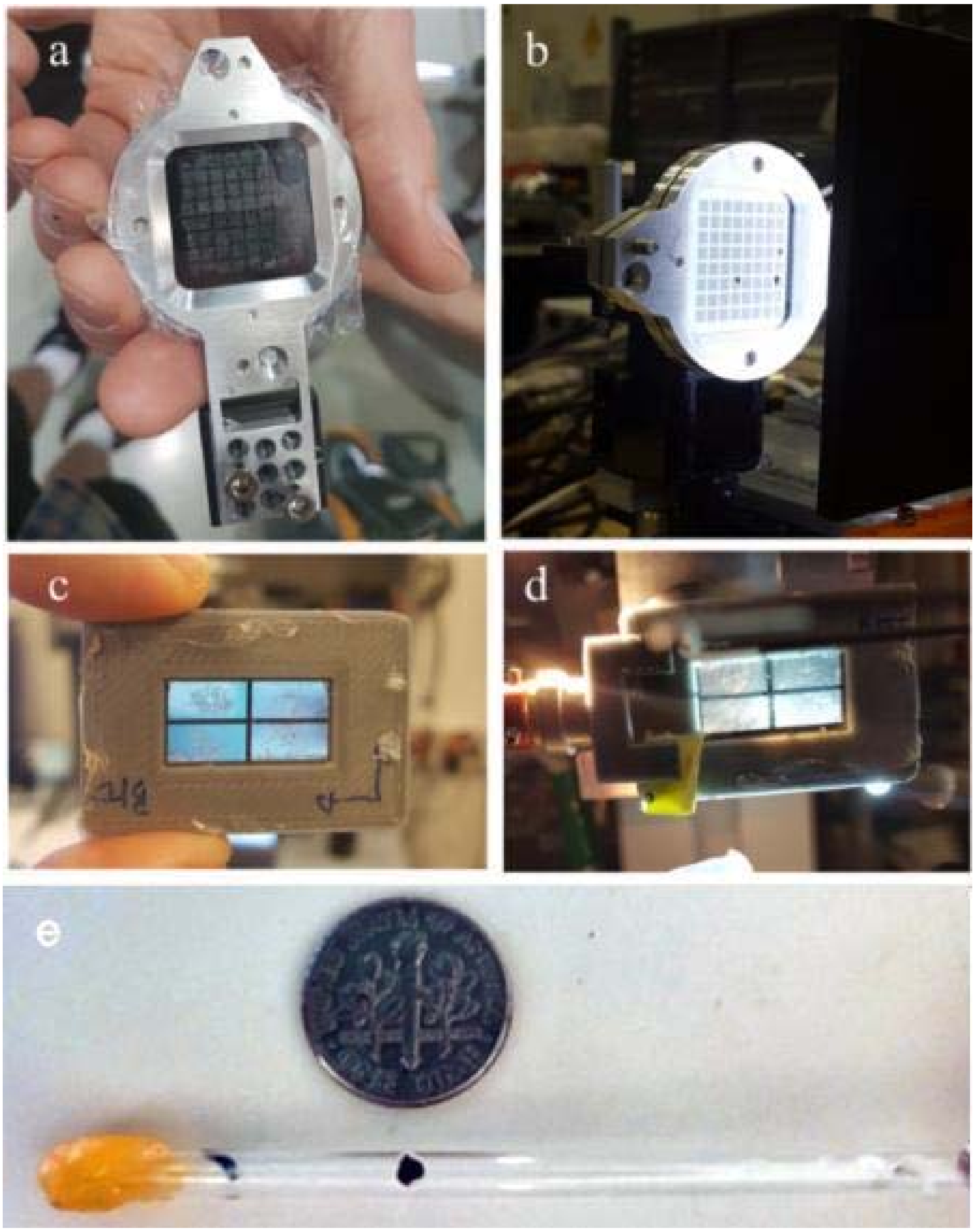

Figure S1 Fixed-target sample delivery methods used for RT crystallography. Images of the silicon nitride chip used for SSX and SFX: **(a)** loaded with crystals and **(b)** mounted in position for X-ray data collection; Images of 'Heidelberg chip' (Doak *et al.*, 2018) used for SLX: loaded with crystals **(c)**, and mounted at the APS BioCARS beamline **(d)**; Image of perdeuterated single crystal mounted in a capillary for NX and NX-Xray with a dime for reference **(e)**.

A novel variety of crystallography "chip" was employed for the SLX measurements. Photos of the chip assembly in use during the SLX measurements are shown in Figure S1 (c) and (d). The exploded view drawing of Figure S2 clarifies the details of the assembly. Unique is the full encapsulation of the chip and the inclusion within the encapsulated cavity of a reservoir saturated with a solution of choice, generally the "mother liquor" in which the crystals have been precipitated. The reservoir, laser-cut from 1 mm thick felt sheets of polyester, rayon, or wool, is saturated with the solvent just before it and the chip are sealed into the holder assembly, yielding a saturated vapor environment around the crystals in which they can remain fully intact for at least several hours. This Sealed Saturated Vapor Environment (SSVE) approach effectively eliminates the common problem of crystal desiccation on crystallography chips, at the cost of somewhat higher background scattering due to the thin polymer films that seal the front and back of the chips. A wide variety of thin polymer films is readily available [1]. By judicious choice of material and thickness of the film, the X-ray background scattering can generally be held within reasonable bounds without sacrificing the desired SSVE attributes of the encapsulation.

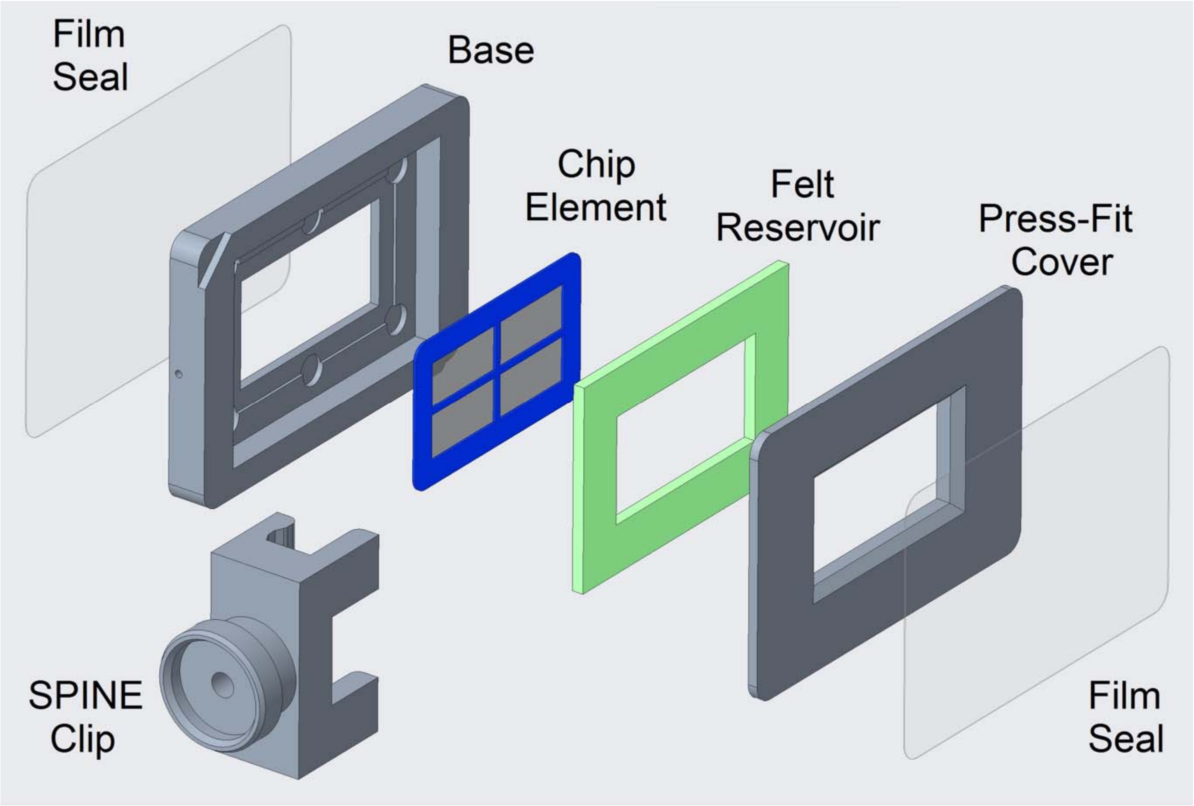

Figure S2 Exploded view of the Sealed Saturated Vapour Environment crystallography chip assembly.

The SSVE chip incorporates several other novel features:

Firstly, although the chip element is fabricated from silicon using standard photolithographic processing techniques, the support film on which the crystals rest is not a thin layer of silicon or silicon nitride, as is generally the case, but rather a layer of photoresist. The material, Cyclotene, is a spin-on dielectric often employed in microelectronics fabrication. It is a glassy polymer composed mostly of low-Z hydrocarbons plus a small percentage of nitrogen and oxygen atoms. In contrast to silicon or silicon nitride, the Cyclotene layer accordingly gives rise to much less X-ray background scattering. Moreover, being glassy, it avoids complications due to X-ray diffraction from the crystal support film. Fabrication of the SSVE chips is straightforward: Cyclotene is spun onto a 30 µm thick silicon substrate to a thickness of 5 µm, cross-bonded, and the silicon then completely etched away down to the photoresist layer to leave 5 µm thick Cyclotene windows of chosen dimensions, separated and supported by an outer rim and cross-bars of silicon. A desired pattern of through holes is etched through the Cyclotene as detailed below. The chips, of overall outer dimensions 15 mm x 25 mm, were fabricated with either four windows measuring 4.67 mm x 9.67 mm each and separated by 0.66 mm wide silicon bars, or six windows measuring 4.53 mm x 6.13 mm each and separated by 0.65 mm bars. The overall window pattern is centered on the chip in both cases. Four different dual hole patterned arrays were fabricated, (i) 7 µm square plus 15 µm square holes, (ii) 10 µm plus 20 µm, (iii) 20 µm plus 30 µm, and (iv) 20 µm plus 40 µm. The nearest-neighbour spacing of the holes was 75 µm in all cases, which was deemed adequate to avoid damage to crystals in neighbouring holes during X-ray exposure of any specific hole. To date the 7 µm plus 15 µm hole pattern has proven to be the most popular.

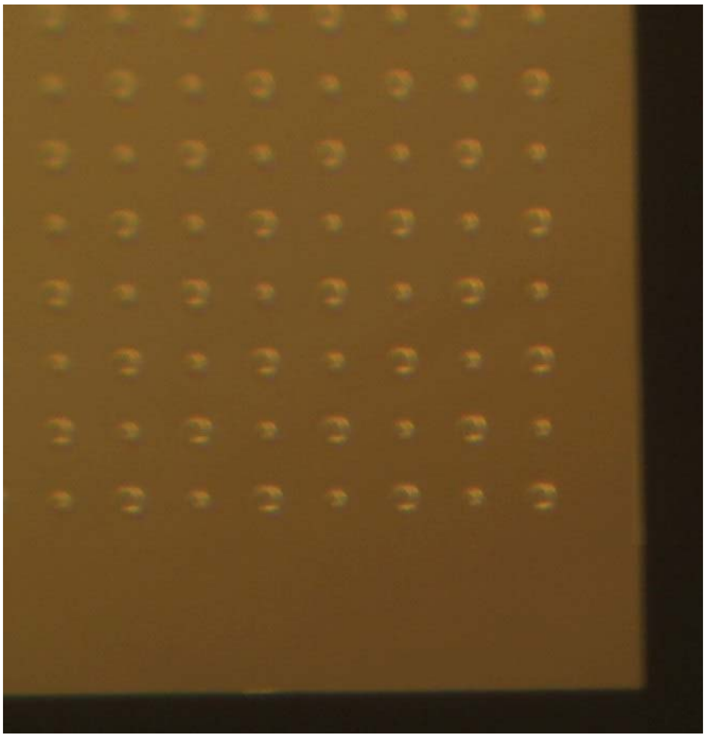

Figure S3 Microscope image recorded near a window corner of a 6-window chip with 7 µm and 15 µm square through-holes at a nearest neighbor spacing of 75 µm between adjacent holes.

Additionally, the base and cover of the SSVE holder are 3D-printed from polylactic acid (PLA), a standard and biocompatible 3D printing material. The chosen sealing films are attached to the base and cover using double-sided tape. The chip element is loaded with sample and blotted, then placed gently into a slight recess within the larger recess of the base piece. Slight circular cut-outs around the periphery of this recess facilitate insertion and removal of the chip element. The felt "sponge" is saturated with the chosen liquid and placed into the larger recess. A rectangular boss on the inside of the cover inserts into the larger recess on the base piece, compressing the felt reservoir slightly against the rim of the chip to both improve the encapsulation and to gently press and firmly hold the SSVE chip into its recess. The cover presses into the base with a tight, low leakage close press-fit that also enables rapid, easy, tool-free assembly of the parts. External notches on two opposing corners of the base allow the cover to easily be pried off and separated from the base. Neither the Cyclotene support film nor the two sealing films are damaged by exposure to X-rays under typical synchrotron conditions. The cover can therefore be removed, the felt reservoir and SSVE chip element removed, and the chip element carefully cleaned for re-use. The chip element should not be allowed to dry before cleaning. Simple rinsing with water or an appropriate solvent is the preferred cleaning technique. Sonication will fracture the Cyclotene layer. The chip element must be handled carefully with forceps, grasping it

only by its silicon frame. When placed on any flat surface, a chip element must be positioned with its recessed side facing downwards to avoid surface tension adhesion that can otherwise tear the Cyclotene layer from the silicon frame when the chip is subsequently lifted.

Finally, as seen in Figure S1 and indicated in S2, the encapsulated chip presses into a 3D-printed PLA clip that is screwed to a commercial SPINE-style magnetic goniometer base [2]. Since the entire assembly weighs very little, the force of the magnetic button attachment suffices to hold the assembly firmly in place on the goniometer. The inner faces of the PLA clip – cylindrical on one side, planar on the other – nominally position the face of the SSVE chip exactly parallel to the axis of the goniometer. To access all 120 x 60 = 7200 holes (half of them large holes, half small) in a single window of a 4-window SSVE chip, the required raster scan is 8.93 mm along the long direction of the chip and 4.43 mm along the short direction. By repositioning the 4-window SSVE chip in the SPINE clip (sliding transversely and/or flipping the chip), the remaining three windows of that chip element can then be accessed with the same goniometer translations. For the 6-window chip the corresponding values for a raster scan of a single window (80 x 56 = 4480 holes) are 5.93 mm and 4.13 mm. To scan across all window area on a SSVE chip without repositioning the chip in the SPINE clip, the goniometer head must translatable over a 20 mm x 10 mm region (for the 4-window chip, 28,800 holes) or a 9.70 mm x 19.68 mm region (6-window chip, 26,880 holes). A similar constraint is common to all crystallography chips, of course.

The SSVE chip system was also tested at an XFEL. The results were not encouraging, although it should be noted that a conventional silicon chip also performed very poorly during the same run under the XFEL pulse energy, duration, and frequency in use [3] That XFEL run, incidentally, led to the spur-of-the-moment invention of the aptly named SOS chip [3], which performed admirably to salvage the beam time [3]. The XFEL difficulties of the SSVE design arise through the extraordinarily intense XFEL pulse, which can vaporize appreciable quantities of any matter exposed to it. In a conventional chip experiment or a conventional liquid jet injection experiment, the vaporized material flies radially outward to deposit on the distant walls and components of the XFEL scattering chamber. Within the encapsulated SSVE cavity, however, the vaporized material deposits primarily and broadly on the adjacent front and back polymer sealing films. The accumulation of vaporized material on the films is cumulative with number of XFEL pulses. When sufficient material has gathered, it can absorb enough energy from an XFEL pulse to rupture the polymer film. In contrast, the much lower power density in a typical synchrotron beam does not vaporize either the crystals or the films, so this damage process

is non-extant. Given that SOS approach has solved the XFEL damage problems while retaining the environmental encapsulation, it would seem that the future of the SSVE chips lies at synchrotrons.

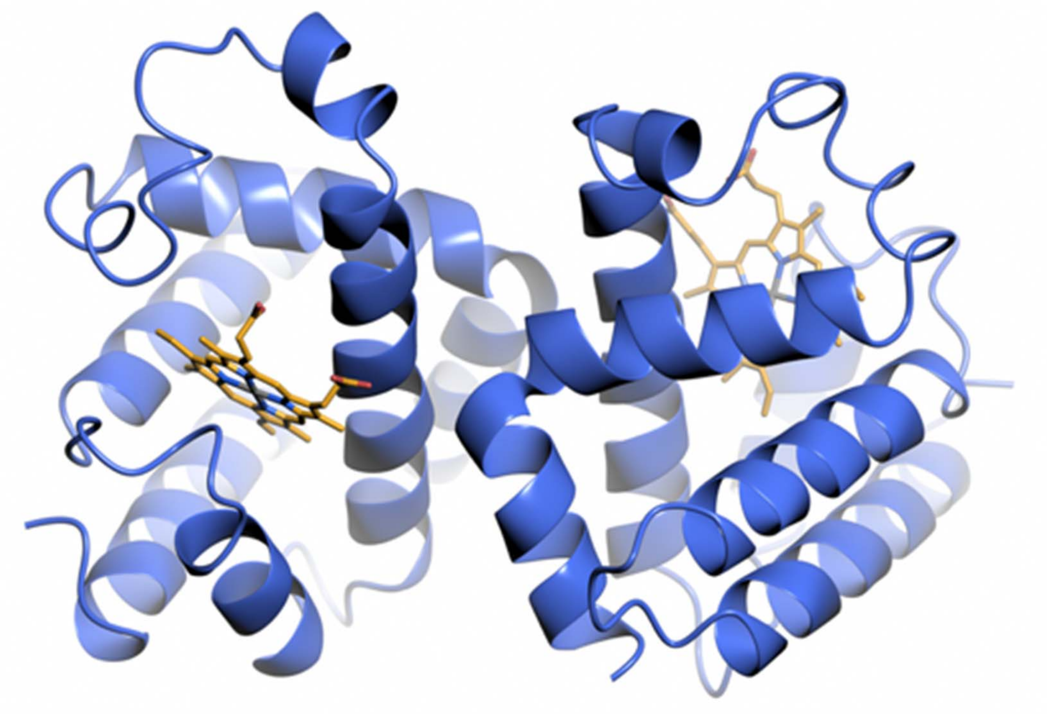

Figure S4 Overall DHP-B structure (model shown is the SFX structure) shows the largely α-helical globin fold of each protomer. Heme groups are shown as sticks.

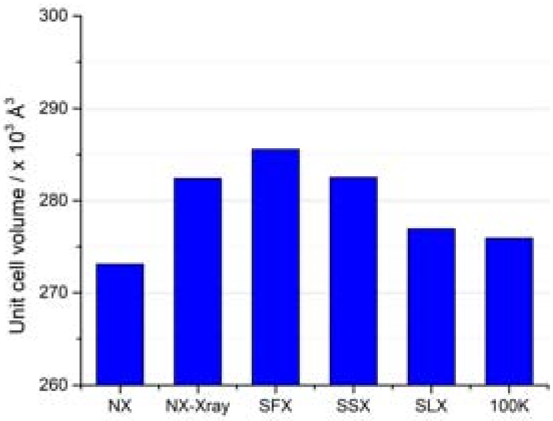

Figure S5 Unit cell volumes (Å^3) for the structures. The value for a 100 K structure of DHP-B (PDB 3ixf) is shown for comparison.

MPEG Titration Studies. Titrations of ferric DHP-B (10 µM) with MPEG 2,000 and MPEG 5,000 (0-250 equiv) in 25 mM cacodylate buffer containing 200 mM ammonium sulfate (components of the crystallization cocktail) were monitored using a Cary 50 UV-vis spectrophotometer at RT as per published protocols (Barrios *et al.*, 2014, Chenprakhon *et al.*, 2010) **(a)**. Constant enzyme and buffer concentrations were maintained against a reference spectrum containing the appropriate amount of MPEG. The titration data showed significant changes to both the Soret and Q-band regions (450-700 nm). Specifically, the addition of MPEG 2,000 to DHP-B [UV-visible: 407 (Soret), 506, 535 (sh), 633 nm] yielded a new spectrum [409 (Soret), 507, 543 (sh), 580 nm; Figure SXA] with a ~25% hypochromicity of the Soret band, a new feature observed at 580 nm, and the loss of the charge transfer band at 633 nm. When titrated with MPEG 5,000, these changes were even more pronounced [413 (Soret), 544, 582 nm **(b)**, with a ~35% hypochromicity and 6 nm bathochromic shift of the Soret band, virtually complete loss of the 506 nm and 633 (charge transfer) features, and the observation of two new bands at 544 and 582 nm. While these changes are not consistent with a previously observed single pure state of DHP-B, they are indicative of substrate-binding induced changes that provide spectroscopic evidence for the formation of the DHP-B:MPEG adduct observed crystallographically.

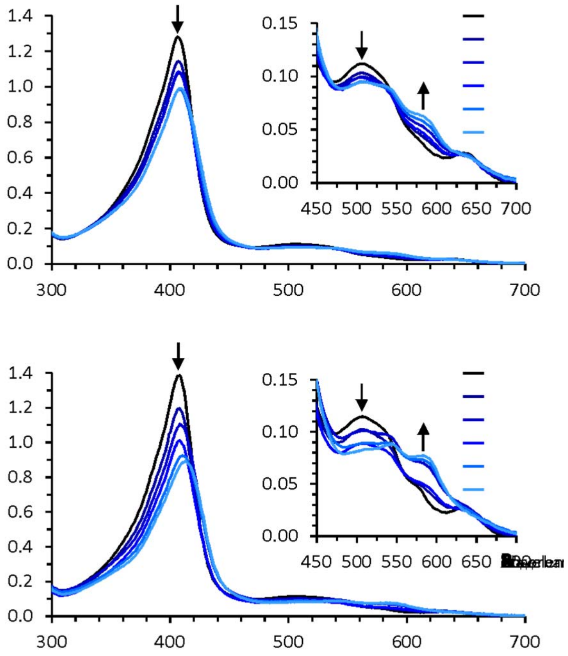

Figure S6 UV-visible spectroscopic monitoring of the titration of 10 µm ferric DHP-B with 0-250 equivalents **(a)** MPEG-2000 and **(b)** MPEG-5000 performed in 25 mM cacodylate buffer (pH 6.5) containing 200 mM ammonium sulfate.

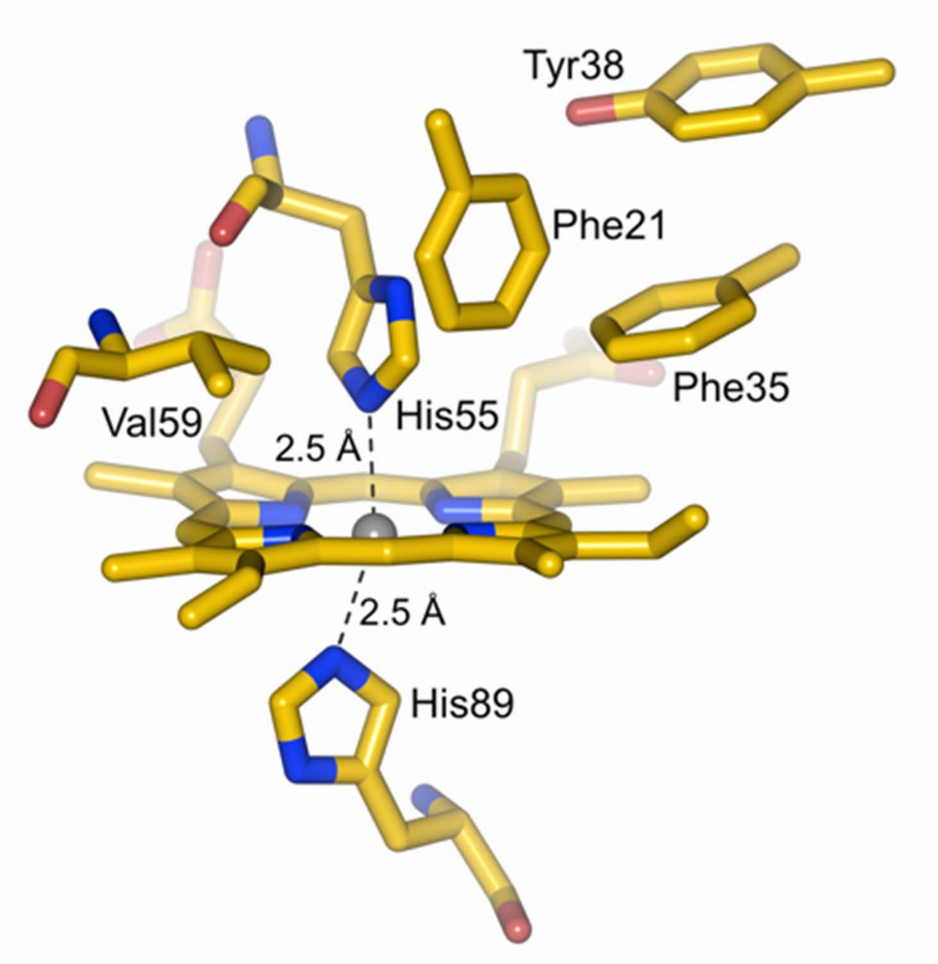

Figure S7 Overall view of the distal site environment for protomer B of the DHP-B neutron crystallography (NX) structure, showing a fully occupied hemichrome species in this protomer. Relevant residues and coordination distances with the heme iron are indicated.

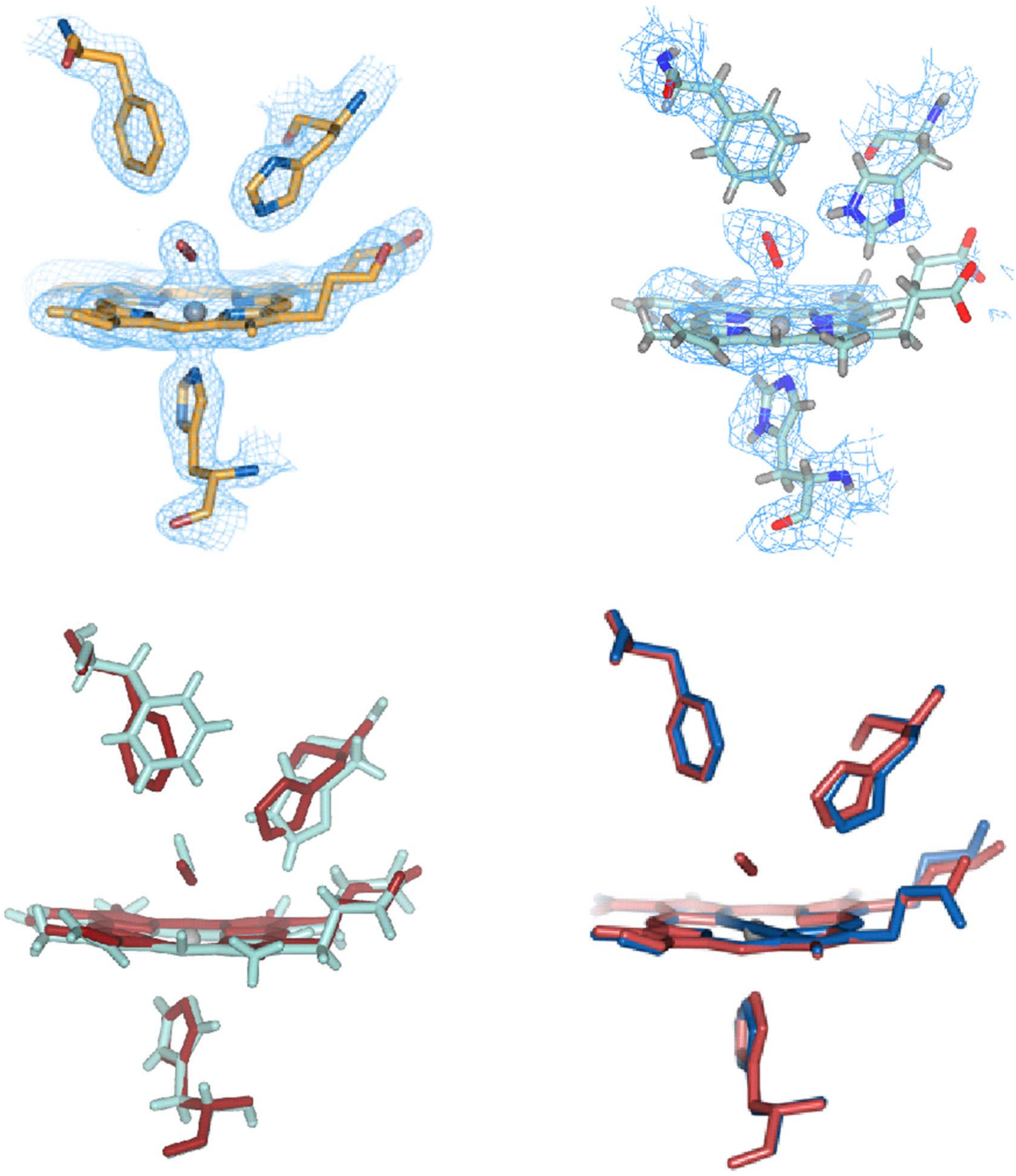

Figure S8 *2Fo-Fc* electron density for the oxyferrous complex of DHP-B from SFX **(a)** and *2Fo-Fc* nuclear density from NX **(b)**. Superposition **(c)** of the NX (green) and SFX (red) structures of oxyferrous DHP-B and **(d)** of the SFX oxyferrous (red) and ferric (blue) structures. Hydrogen atoms are indicated for neutron structures only where they are explicitly modelled based on experimental data. The proximal His 89 residue together with distal Phe 21 and the proximal His 55 are shown in all panels.

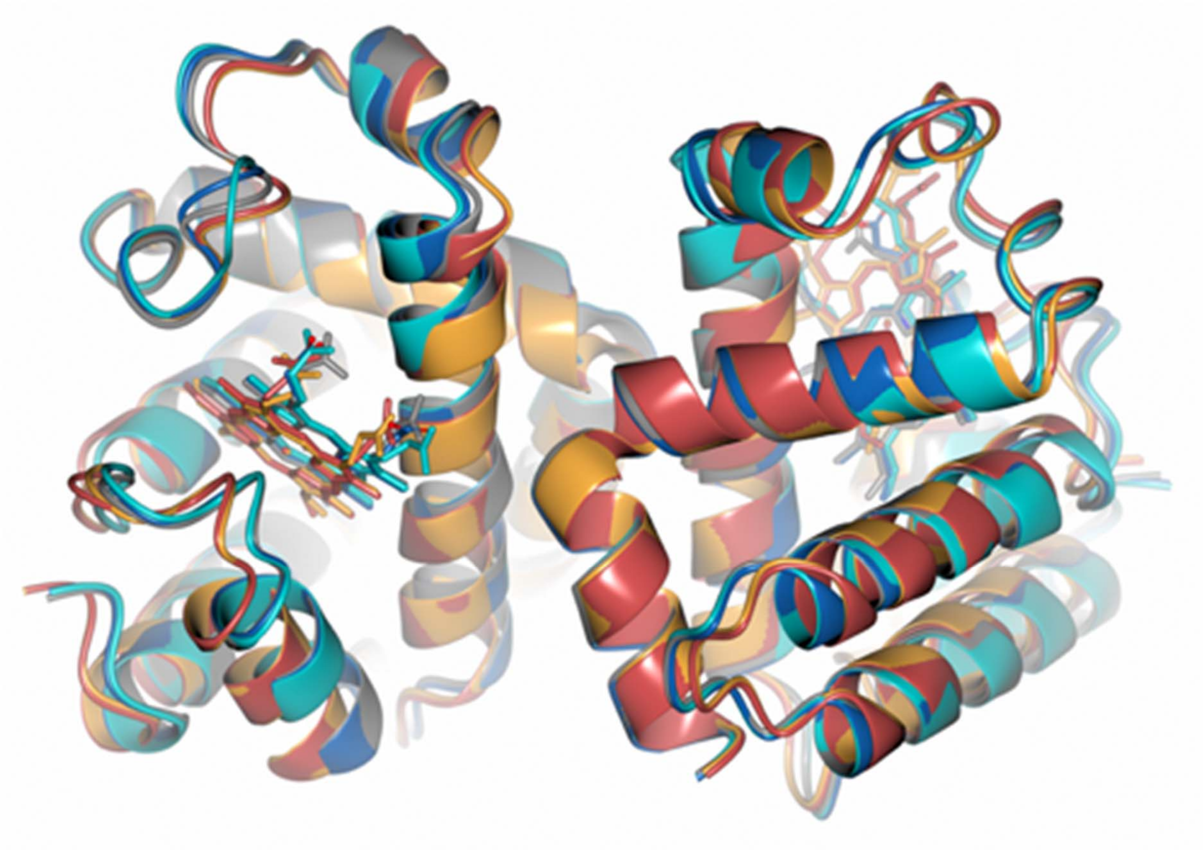

Figure S9 Superposition of the different overall ferric DHP-B structures obtained by SFX (in blue), SSX (in turquoise), SLX (in grey), NX (in orange), and NX-Xray (in red).

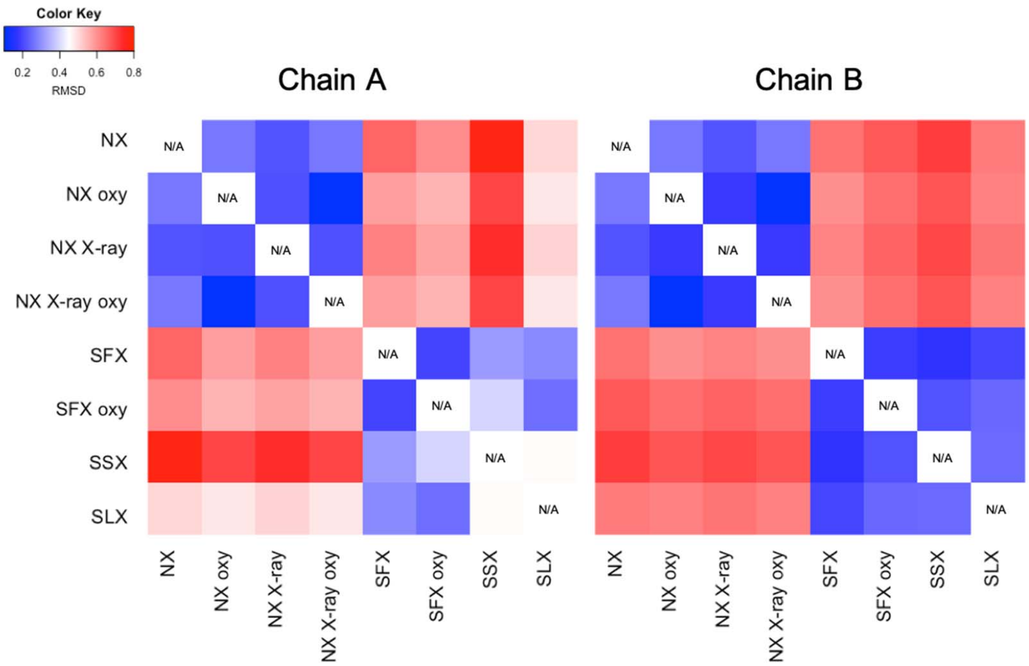

Figure S10 Comparison of Cα RMSD for chains A and B of the DHP-B structures. Distinct clustering of RMSD is notable between the set of neutron structures (NX, NX oxyferrous, NX X-ray and, NX X-ray oxyferrous) and between the set of serially obtained structures (SFX, SFX oxyferrous, SSX and SLX).

References

Barrios, D. A., D'Antonio, J., McCombs, N. L., Zhao, J., Franzen, S., Schmidt, A. C., Sombers, L. A. & Ghiladi, R. A. (2014). *J Amer Chem Soc* **136**, 7914-7925.

Chenprakhon, P., Sucharitakul, J., Panijpan, B. & Chaiyen, P. (2010). *J Chem Education* **87**, 829-831.

Doak, R. B., Nass Kovacs, G., Gorel, A., Foucar, L., Barends, T. R. M., Grunbein, M. L., Hilpert, M., Kloos, M., Roome, C. M., Shoeman, R. L., Stricker, M., Tono, K., You, D., Ueda, K., Sherrell, D. A., Owen, R. L. & Schlichting, I. (2018). *Acta Cryst* **D74**, 1000-1007.
